# Neuroimaging alterations of the suicidal brain and its relevance to practice: an updated review of MRI studies

**DOI:** 10.3389/fpsyt.2023.1083244

**Published:** 2023-04-27

**Authors:** Matthew Dobbertin, Karina S. Blair, Erin Carollo, James R. Blair, Ahria Dominguez, Sahil Bajaj

**Affiliations:** ^1^Multimodal Clinical Neuroimaging Laboratory (MCNL), Center for Neurobehavioral Research, Boys Town National Research Hospital, Boys Town, NE, United States; ^2^Child and Adolescent Psychiatric Inpatient Center, Boys Town National Research Hospital, Boys Town, NE, United States; ^3^Program for Trauma and Anxiety in Children (PTAC), Center for Neurobehavioral Research, Boys Town National Research Hospital, Boys Town, NE, United States; ^4^Stritch School of Medicine, Loyola University Chicago, Chicago, IL, United States; ^5^Child and Adolescent Mental Health Centre, Mental Health Services, Copenhagen, Denmark

**Keywords:** suicidal thoughts and behavior, depression, review, clinical relevance, task-based fMRI, resting state fMRI, brain morphometry, diffusion tensor imaging

## Abstract

Suicide is a leading cause of death in the United States. Historically, scientific inquiry has focused on psychological theory. However, more recent studies have started to shed light on complex biosignatures using MRI techniques, including task-based and resting-state functional MRI, brain morphometry, and diffusion tensor imaging. Here, we review recent research across these modalities, with a focus on participants with depression and Suicidal Thoughts and Behavior (STB). A PubMed search identified 149 articles specific to our population of study, and this was further refined to rule out more diffuse pathologies such as psychotic disorders and organic brain injury and illness. This left 69 articles which are reviewed in the current study. The collated articles reviewed point to a complex impairment showing atypical functional activation in areas associated with perception of reward, social/affective stimuli, top-down control, and reward-based learning. This is broadly supported by the atypical morphometric and diffusion-weighted alterations and, most significantly, in the network-based resting-state functional connectivity data that extrapolates network functions from well validated psychological paradigms using functional MRI analysis. We see an emerging picture of cognitive dysfunction evident in task-based and resting state fMRI and network neuroscience studies, likely preceded by structural changes best demonstrated in morphometric and diffusion-weighted studies. We propose a clinically-oriented chronology of the diathesis-stress model of suicide and link other areas of research that may be useful to the practicing clinician, while helping to advance the translational study of the neurobiology of suicide.

## 1. Introduction

The World Health Organization (WHO) reports that suicide is the second leading cause of death among individuals aged 15–29 years (1) with an estimate of approximately 800,000 people dying from suicide each year - a global mortality rate of one person every 40 s. In the United States, the Center for Disease Control and Prevention (CDC) reports Suicide is the 12th leading cause of death for both Hispanic and non-Hispanic people of all races (2). In 2020, suicide was the second leading cause of death for children (age 10–14 years, accounting for 581 deaths) and third leading cause of death for young individuals (age 15–24 years, accounting for 6,062 deaths) (3). Suicide research has been very important in developing clinical suicide risk assessments (4). However, recent neuroimaging work with suicidal patients holds significant promise for the clinician to directly access information without it being filtered through the situation, suspicion or in fact impaired processes a suicidal individual may use to disclose the risk assessment information. This has been accelerated by parallel theories in neurobiology and cognitive neuroscience (5–7) and a clinical focus from the likes of McGirr (8) and Turecki (9). Recent reviews (10, 11) have also contributed to bridging this translational gap. In spite of this, there still is not a clinically-accessible link between witnessed patient histories, symptoms, and deficits and what is rapidly being discovered in the fields of functional, network, and morphometric neurobiology.

Multiple general theories have been developed in an attempt to understand the risk factors, thoughts, distortions, cognitive/behavioral differences, atypical brain structures, systems and functions that distinguish suicidal individuals from those without past attempts, or a high risk. As clearly laid out in Van Heeringen’s book “the Neuroscience of Suicidal Behavior,” (6) five main neurobiological theories precede the recent work of Schmaal, Auerbach, and others. These include the “Cry of Pain” model (12), the Interpersonal model (7), the Integrated Motivational model (13), the Clinical Stress-Diathesis model (8, 14), and Jollant’s Neurocognitive model (5). The most relevant to this paper is Van Heeringen’s Neurobiological model (6). This model synthesizes Molecular, Morphological, Cognitive, and Functional evidence into a theory based on predictive coding, also known as computational psychology. This model suggests that the main difference between suicidal individuals and those that are not suicidal, have an impaired system of evaluating old beliefs, evaluating the importance and certainty (precision) of new information, and appropriately changing current beliefs and strategies accordingly. Author states that learning is directly affected, proposing that humans are generally biased toward positive valenced stimuli and predictions. However, those with abnormal serotonin systems, which play a role in learning and extinction of behaviors that lead to aversive events, may be biased toward learning more negatively valenced behaviors (called Pavlovian instrumental transfer). Author further proposes that belief updating (modifying old beliefs based on new sensory information) is dependent on the right inferior frontal gyrus (IFG) and bilateral superior frontal gyrus (SFG) for positive valenced information, and on the left IFG and right inferior parietal lobule (IPL) for negative information. Lastly, as with disrupted serotonin and NMDA systems, blunted cortisol reactivity to stress creates a founding diathesis and may account for the higher rates of suicidality amongst individuals with trauma and adverse childhood events.

Our goal is to consider the current body of neuroimaging research on STBs (defined here as suicidal ideation and attempts) among those with Major Depressive Disorder (MDD) (15). Our goal is to clarify relevant concepts for the clinicians working with these patients and to propose the framework of a testable timeline of the suicidal brain in this population that may be developed into a clinical tool.

## 2. Methods

A search on functional, structural, and diffusion-weighted MRI studies of the suicidal brain was performed in the search engine ‘PubMed’ for both original and review research articles that were published before December 2021. The literature search was conducted using the following terms in the title:

*((BOLD[Title]) OR (fMRI[Title]) OR (functional MRI[Title]) OR (functional magnetic resonance imaging[Title]) OR (morphometry[Title]) OR (thickness[Title]) OR (surface area[Title]) OR (volume[Title]) OR (gyrification[Title]) OR (folding[Title]) OR (MRI[Title]) OR (DTI[Title]) OR (magnetic resonance imaging[Title]) OR (diffusion tensor imaging[Title]) OR (brain connectivity[Title]) OR (connectivity[Title]) OR (brain activation[Title]) OR (neural activation[Title]) OR (neuroimaging[Title]) OR (spectroscopy[Title]) OR (white-matter[Title]) OR (gray-matter[Title]) OR (neural correlates[Title]) OR (neural representations[Title])) AND ((suicide[Title]) OR (suicidal[Title]) OR (suicidality[Title]) OR (suicide risk[Title]) OR (self-harm[Title]) OR (suicidal ideation[Title])) NOT ((Inflammation[Title]) OR (Inflammatory[Title]) OR (Plasma[Title]) OR (Tumor[Title]) OR (Immunity[Title]) OR (habenula[Title]) OR (mRNA[Title]) OR (RNA[Title]) OR (DNA[Title]) OR (Gene[Title]) OR (Genetics[Title]) OR (Glucose[Title]) OR (Brain expression[Title]) OR (Pain[Title]))*. The search was performed without a time limit. This resulted in a total of 149 articles. The articles were further filtered through an inspection of the abstracts. A total of 80 articles that included studies focusing on patients with clinical conditions other than MDD, studies focusing on non-suicidal self-injury, and studies relating to neuroimaging modalities other than MRI (i.e., MEG, PET) were excluded.

## 3. Results

A total of 69 research articles, focusing on STBs associated with depression, were identified, and reviewed in the current study (Tables 1–4).

**Table 1:** Task-based fMRI (TBfMRI) studies.

Author	Mode	Task	Findings	Population
Brown et al. (2020)	TBfMRI	Decision Making	↓ BOLD in vmPFC activity in SUIATT and impulsivity not correlated to vmPFC-FP connectivity ➔ compared to HC	Adults with and without STB (past attempts, ideation only) vs. HC
Li et al. (2020)	TBfMRI	Iowa Gambling Task (IGT), Tower of London Task, Go/No-Go task, Faces and Shapes fMRI task, and Emotion-Processing Task	↓ BOLD in fusiform gyrus but ↑ in left insula activation in SUIATT	Adolescent and adult MDD + SUIATT vs. MDD alone
Olié et al. (2017)	TBfMRI	Cyberball Game/Task	↓ BOLD in left insula and SMG in SUIATT vs. both controls	36 Women with MDD + STB vs. 41 with history of MDD vs. 28 HCs
Cáceda et al. (2020)	TBfMRI	Cyberball Game/Task	No group differences in activation, but with social exclusion: 1. Suicide risk correlated with BOLD in superior insula 2. Depression severity and psychological pain correlated with BOLD in superior insula 3. BOLD in dACC correlated with physical pain severity	Adults with MDD and SUIATT vs. MDD + SI vs. MDD vs. HCs
Miller et al. (2018)	TBfMRI	Facial Affective Task requiring regulation of response before stimuli presentation	↑ BOLD in dlPFC among SUI vs. HCs	Adolescents with SI vs. HCs
Davis et al. (2014)	TBfMRI	Emotion Regulation-Reappraisal	↑ BOLD in amygdala vs. controls	Adults with STB (no distinction) vs. Adults with Depression and Anxiety without STB vs. HCs
Jollant et al. (2008)	TBfMRI – Passive Task	Visual Affective Valence Task	↑ BOLD in lateral OFC and ↓ BOLD in SFG during angry stimuli, ↑ BOLD ACC to happy stimuli, and ↑ BOLD in cerebellum to mild angry stimuli in the MDD + STB group	Adult men with MDD + STB vs. MDD vs. HCs
Richard-Devantoy et al. (2016)	TBfMRI	Go/No-Go Response Inhibition	No difference between SUIATT and controls; No association between SUIATT and BOLD	Adults with MDD + SUIATT vs. MDD vs. HCs
Pan et al. (2013)	TBfMRI	Emotionally Valenced Gender Discrimination Task	SUIATT ↑ BOLD in right ACC, bilateral primary sensory cortex, left dlPFC, and right MTG during angry faces; SUIATT ↓ BOLD in left fusiform gyrus during neutral faces compared to MDD. ↑ BOLD in primary sensory cortex during angry compared to HCs	Adolescents with SUIATT + MDD vs. MDD vs. HCs
Ai et al. (2018)	TBfMRI	Emotionally Valenced Gender Discrimination Task	SUIATT ↓ BOLD in fusiform gyrus across all emotional valences vs. controls	Adults with SUIATT + MDD vs. SI + MDD vs. MDD
Alarcón et al. (2019)	TBfMRI/FC	Emotional Self-Face Recognition Task	↑ FC between amygdala and dlPFC, dmPFC, and precuneus in SUIATT + MDD vs. HCs	Adolescents with SUIATT + MDD vs. high SI + MDD vs. low SI + MDD vs. HCs
Malhi et al. (2019)	TBfMRI/FC	Emotional Face-Word Stroop Task	↑ BOLD in PFC, frontopolar cortex, ACC, and posterior parietal cortex; and ↑ activity among basal ganglia structures with increasing suicide risk	Adults with STB (both SI and SUIATT) + Mood Disorder vs. HCs
Just et al. (2017)	TBfMRI Machine Learning	Neurosemantic (Presentation of words associated with life and death)	Areas found to be significantly associated with suicidal ideation = medial superior frontal, inferior parietal, medial temporal, anterior cingulate, and inferior frontal cortices	SUI vs. HCs

**Table 2:** Resting-state fMRI studies.

Author	Mode	Findings	System interactions	Population
Qiu et al. (2020)	RSFC	↑ SI = ↓ RSFC between pregenual ACC and SFG	↑ SI = ↓ between**M-CIN/SN** and**M-FPN/DMN**	Adults with MDD + SUIATT vs. MDD
Du et al. (2017)	RSFC	↓ RSFC between ACC (**M-CIN/SIN**) and orbito-medial PFC (**M-FPN/DMN**) in MDD + SI	↓ RSFC between**M-CIN/SN** and**M-FPN/DMN**	Adults with MDD + SI vs. MDD vs. HCs
Yang et al. (2020)	RSFC/Morphometric	↓ RSFC in right inferior Orbitofrontal gyrus; ↓ GMV in right IFOG and left caudate	↓RSFC between**M-FPN/DMN** and**M-CIN/SN**	Adults with MDD + SUIATT vs. MDD
Stange et al. (2019)	RSFC	↓ RSFC in rMFG/SFG and**M-FPN/DMN;** ↓ RSFC between precuneus and**M-CIN/SN**	↓ RSFC between**L-FPN** and**M-CIN/SN**, ↓ RSFC between**L-FPN/CCN** and**M-FPN/DMN**	Adults with Mood Disorders + SUIATT vs. Mood Disorders vs. HCs
Cao et al. (2016)	RSFC/Low Frequency Resting Activation	↑ fALFF in right STG, left MTG, and left MOG	*N/A	Adolescents and young adults with MDD + SUIATT vs. MDD vs. HCs
Cao et al. (2021)	RSFC	↓ RSFC between left MFG and left SPG	↓ RSFC between**L-FPN/CCN** and left**D-FPN/AN**	Young adults with MDD + SUIATT vs. MDD vs. HC
Zhang et al. (2020)	RSFC	↑ RSFC between bilateral amygdala and bilateral paracentral lobule/precuneus in SUIATT and SI vs. HCs	↑ RSFC between**M-FPN/DMN** and**M-CIN/SIN** in STB groups vs. HCs	Adolescents and young adults with Mood Disorders + SUIATT vs. Mood Disorders + SI vs. Mood Disorders vs. HCs
Kang et al. (2017)	RSFC	↑ RSFC from left amygdala to the right insula and left superior OFC and increased FC of the right amygdala with the left middle temporal area	↑ RSFC between**M-CIN/SN** to**M-CIN/SN** and**M-FPN/DMN**	Adults with MDD + SUIATT vs. MDD
Wei et al. (2018)	RSFC	↑ RSFC amygdala to precuneus/cuneus compared to non-suicidal and HCs	↑ RSFC between**M-CIN/SN** to**M-FPN/DMN**	Adults with MDD + SI vs. MDD vs. HCs
Cao et al. (2020)	RSFC	↓ RSFC between (superior frontal gyrus and medial frontal gyrus) and (bilateral anterior insular and anterior cingulate cortices, and the temporal–parietal junction area); ↑ RSFC between (bilateral anterior insular and anterior cingulate cortices and the temporal–parietal junction area) and (precuneus, inferior parietal lobule, middle frontal gyrus, and superior parietal lobule)	↓ RSFC between**M-FPN/DMN** and**L-FPN/CCN**, ↑ RSFC between**L-FPN/CCN → M-CIN/SN**	Adolescents and young adults with MDD + SUIATT vs. MDD vs. HCs
Shu et al. (2020)	RSfALFF	↑ fALFF in posterior cerebellum, right ACC, left caudate and left SFC; ↑ fALFF in left middle occipital cortex and left precuneus after treatment vs. HCs	↑ fALFF within **M-FPN/DMN**, **L-FPN/CCN**, and **M-CIN/SN** in SUIATT; ↑ fALFF in **L-FPN/CCN** after treatment	Adults with SUIATT + MDD vs. HCs
Zhang et al. (2016)	RSFC	↑ RSFC in cerebellum; ↑ between frontal and parietal lobes within **M-FPN/DMN**	↑ RSFC between areas within **M-FPN/DMN** but ↓ RSFC between others within **M-FPN/DMN**	Adolescents and adults with MDD + SUIATT/SI vs. HCs
Chen et al. (2021)	RSFC, Correlation analysis, fALFF, ReHO	In MDD + SI vs. MDD: ↑ RSFC in right and left hippocampus; ↓ fALFF in left cuneus; ↑ fALFF in right MTP; ↓ ReHO in right cuneus; ↑ ReHO in left MTG. In MDD vs. HC: ↓ in right and left thalamus and both right and left MC	↑ RSFC between regions within **M-FPHN**/**DMN**	Adults with MDD + SI vs. MDD vs. HCs
Barredo et al. (2019)	RSFC and Morphometric (cortical thickness)	↑ RSFC in pars orbitalis, striatum, and thalamus = ↑ cortical thickness = ↑ self-reported suicidality	↑ RSFC between regions within **M-FPN**/**DMN**correlates with cortical thickness	Adults with PTSD with scale of varying depression scores vs. HCs
Lee et al. (2019)	RSFC	↑ RSFC between anterior right parahippocampal gyrus to posterior parahippocampal gyrus	↑ RSFC within **M-FPN/DMN** in MDD + SUIATT vs. HCs	Adults with MDD + SUIATT vs. HCs
Schreiner et al. (2019)	RSFC	↑ RSFC between right precuneus and right IFG and cerebellum and between left PCC, left cerebellum, and cingulate gyrus	↑ RSFC between components of **M-FPN/DMN**	Adolescents with MDD with vs. without medication treatment
Kim et al. (2017)	RSFC	↓ RSFC in SFG, pars orbitalis, left thalamus, and right thalamus compared to the whole brain among MDD + SI vs. HCs	↓ RSFC in **M-FPN/DMN** to whole brain in MDD + SI vs. HCs	Adults with MDD + SI vs. MDD vs. HCs
Gosnell et al. (2019)	ML RSFC	↓ RSFC between rSFG and insula; ↑ RSFC between left habenula and right parahippocampus; ↑ RSFC between left MFOG and left Rolandic operculum; ↑ RSFC between left putamen and cerebellar vermis; ↓ RSFC between amygdala and MTP	↓ RSFC in **M-FPN/DMN** and **M-CIN/SN**; ↑ RSFC between **M-CIN/SN** and **M-FPN/DMN**; ↑ RSFC between **M-FPN/DMN** and **L-FPN/CCN**; ↑ RSFC between left putamen and cerebellar vermis; ↓ RSFC between **M-CIN/SN** and **D-FPN/AN**	Adult inpatient psychiatric patients with SI and/or SUIATT vs. without
Dai et al. (2020)	ML RSFC	Significant areas distinguishing high suicide risk = right inferior temporal gyrus, left inferior frontal gyrus, right anterior angular gyrus, left inferior parietal cortex, left Rolandic operculum, and right dorsolateral superior frontal gyrus	Significant areas were found in **L-FPN/CCN**, **M-FPN/DMN** primarily	Adults with MDD + SUIATT/SI vs. MDD
Stumps et al. (2020)	ML RSFC	Right amygdala and right MTG specifically correlated to high suicide risk group	**M-CIN(SN)**, cognitive-control **(L-FPN)**, **M-FPN**, and visual networks	Adults with trauma with SUIATT vs. Clinical vs. Trauma-exposed
Chase et al. (2017)	RSFC	↑ RSFC between dorsal PCC and MTG	↑ RSFC between**M-FPN/DMN** and**D-FPN/**Attn	Adults with MDD + SI vs. HCs
Serafini et al. (2016)	RSFC Review	Activity mixed among networks, no distinction between pathologies	Activity mixed among networks, no distinction between pathologies	Review

**Table 3:** Morphometric studies.

Author	Mode	Findings	Population
Hwang et al. (2010)	Cortical Thickness	Cortical thinning in the left dlPFC, vlPFC, and ACC in MDD + SUIATT	Adults with MDD + SUIATT vs. MDD
Wagner et al. (2012)	Cortical Thickness	Cortical thinning in the left dorsolateral, ventrolateral prefrontal, and ACC in MDD + STBs	Adults with MDD + high risk vs. MDD without high risk for suicide
Huber et al. (2021)	Cortical thickness and volume, RSFC	Cortical thickness of the anterior cingulate/paracingulate cortex was shown to predict the functional connectivity between the lateral pars orbitalis and anterior cingulate/paracingulate	Adult veterans with SUIATT vs. SI
Wang et al. (2020)	GMV	Reduced GMV in left and right MFG among MD + SAs compared to other groups	Adolescents and adults with MDD/BD + SUIATT vs. MDD/BD + SI vs. MDD vs. HCs
Ding et al. (2015)	GMV	↓ GMV in left vlPFC in suicide attempters	Adults with Mood Disorders + STB vs. Mood Disorders vs. HCs
Fan et al. (2019)	Morphometric and DTI	↓ GMV in left vlPFC and left dlPFC; ↑ GMV in the left vlPFC compared to depressed but non-suicide attempters	Adolescents and adults with MDD + SUIATT vs. BD + SUIATT vs.
Lippard et al. (2019)	Morphometric	Lower baseline ventral and rostral prefrontal GMV compared to those who did not attempt	Adolescents and adults with Mood Disorders + SUIATT vs. Mood Disorders + future SUIATT vs. Mood Disorders
Segreti et al. (2019)	Morphometric	↓ GMV in left MFG; ↓ Cortical thickness within the posterior frontal lobe including the bilateral precentral gyrus	Adults with SI vs. HCs
Bajaj et al. (2019)	Morphometric	↓ Cortical surface area and volume within the left dlPFG with ↑ SI	Non-clinical adults
Kang et al. (2020)	Morphometric	↑ CSA in left postcentral and left lateral occipital areas and ↑ CV in left postcentral and left lateral OFC, but ↓ CSA in left SFG among MDD + SUIATT	Adults with MDD + SUIATT vs. MDD
Harenski et al. (2020)	Morphometric	SUIATT ↓ GMV in PCC/precuneus, IPC, dorsal prefrontal cortex, amygdala, insula, superior occipital gyrus, cuneus, and cerebellum	Adult criminal offenders with SUIATT vs. no SUIATT vs. HCs
Kang et al. (2020)	Morphometric	↑ GSA in left postcentral area and left lateral occipital area and a larger CV in the left postcentral area and left lateral orbitofrontal area among SUIATT; ↓ CSA in left superior frontal area than suicide non-attempters	Adults with MDD + SUIATT vs. MDD
Gosnell et al. (2016)	Morphometric	↓ Volume of the right hippocampus	Adults with MDD + SUIATT vs. MD
Chen et al. (2020)	Morphometric and Cell Type Analysis	↑ Neuron number in CA2/3 subregions of the hippocampus gyrus	Post-mortem MDD + suicide vs. MDD vs. Schizophrenia + suicide vs. Schizophrenia vs. HCs
Jollant et al. (2018)	Morphometric	Association between family history of suicide and ↓ volume within the bilateral temporal regions, right dlPFC, and left putamen, as well as between violent method of attempt and increased bilateral caudate and left putamen volumes	Adults with SUIATT vs. Patient Controls vs. HCs
Ho et al. (2018)	Morphometric	↓ GMV in the dorsal striatal structures, particularly bilateral putamen and caudate, were associated with greater implicit SI observed from suicide-related outcomes from the death version of the Implicit Association Test	Adolescent Clinical vs. HCs
Ho et al. (2021)	Morphometric	↓ GMV in the dorsal striatal structures, particularly bilateral putamen and caudate, were associated with greater implicit SI observed from suicide-related outcomes from the death version of the Implicit Association Test	Adolescent Clinical vs. HCs
Pan et al. (2015)	Morphometric	↓ GMV in right STG	Adolescents with MDD + SUIATT vs. MDD
Vidal-Ribas et al. (2021)	Morphometric	↓ GMV in superior temporal sulcus in children aged between 9 and 10 years	Children with no previous diagnosis or STB
McLellan et al. (2018)	Morphometric	↓ of the right STG in adolescents with MDD	Adolescents and adults with MDD(TRD) + SUIATT vs. MDD(TRD) vs. HCs
Peng et al. (2014)	Morphometric	MDD + SUIATT showed ↓ GMV within the right MTG and ↑ GMV within the right parietal lobe vs. HCs	Adults with MDD + SUIATT vs. MDD vs. HCs
Lee et al. (2016)	Morphometric	↓ GMV in the left anterolateral region of the parietal lobe as well as in the right cerebellum in MDD + SUIATT	Adults with MDD + SUIATT vs. MDD
Campos et al. (2021)	Morphometric	↓ GMV of thalamus and right pallidum significantly smaller in MDD + SUIATT vs. MDD and HCs; ↓ CSA of the left cuneus, left inferior parietal, left rostral middle frontal, and right pericalcarine cortex in MDD + SUIATT vs. HCs; MDD + SUIATT ↓ Cortical thickness in left rostral middle frontal cortex	Adults Enigma Metanalysis MDD + SUIATT vs. MDD vs. HCs
Sarkinaite et al. (2021)	Morphometric	↓ Thickness of temporal cortex in inferior middle and temporal cortex as number of SUIATT ↑	Hospitalized adults with first SUIATT vs. > 1 SUIATT vs. HCs

**Table 4:** Diffusion tensor imaging (DTI) studies.

Author	Mode	Findings	Population
Jia et al. (2010)	DTI	↓ FA in left anterior limb of the internal capsule (ALIC) among MDD + SUIATT; ↓ FA in right frontal lobe vs. HCs, and ↓ FA in right lentiform nucleus (putamen) vs. MDD	Adults with MDD + SUIATT vs. MDD vs. HCs
Jia et al. (2014)	DTI	MDD + SUIATT showed ↓ MPF from the ALIC to the left medial frontal cortex, left OFC, and left thalamus.	Adults with MDD + SUIATT vs. MDD vs. HCs
Kim et al. (2015)	DTI	↑ FA among PD + SA vs. PD in retrolenticular part of the internal capsule, splenium of the corpus callosum, superior and posterior corona radiata, posterior thalamic radiations, sagittal stratum (including the inferior longitudinal fasciculus and inferior fronto-occipital fasciculus), and superior longitudinal fasciculus	Adults with PD + SUIATT vs. PD vs. HCs
Myung et al. (2016)	DTI	MDD + SI had ↓ SC/EW across cortical (i.e., rostral middle frontal cortex, superior parietal cortex, subdivisions of the inferior frontal cortex [pars triangularis and pars orbitalis, frontal pole, and lateral occipital cortex]) and subcortical (i.e., pallidum, thalamus, putamen, and caudate) regions in the left hemisphere	Adults with MDD + SI vs. MDD vs. HCs
Olvet et al. (2014)	DTI	↓ FA among MDD + SUIATT vs. MDD vs. HCs in right dmPFC and white-matter bundles in several regions including the bilateral inferior fronto-occipital fasciculus, bilateral uncinate fasciculus, body of corpus callosum, right anterior limb of internal capsule, right external capsule, left posterior thalamic radiation, and bilateral posterior corona radiata	Adults MDD + SUIATT vs. MDD vs. HCs
Wei et al. (2020)	DTI	↓ FA among MDD + SUIATT compared to non-attempters with MDD and healthy controls in right dmPFC and bilateral inferior fronto-occipital fasciculus, bilateral uncinate fasciculus, body of corpus callosum, right anterior limb of internal capsule, right external capsule, left posterior thalamic radiation, and bilateral posterior corona radiata	Adults with MDD + SUIATT vs. BD + SUIATT vs. MDD vs. BD vs. HCs
Bijttebier et al. (2015)	DTI	↓ Structural connectivity between left olfactory cortex and left anterior cingulate gyrus; ↓ connectivity between the right medial orbital, SFG and the right rectal gyrus, and between the right calcarine fissure and both the left superior and middle occipital gyrus	Adults with MDD + SUIATT vs. MDD vs. HCs
Hwang et al. (2018)	DTI	↑ Edge weight in the left PCC and ↑ structural connectivity of local connections among MDD + SUIATT vs. MDD + SUI	Adult veterans with SUIATT vs. SUI
Chen et al. (2021)	DTI	↓ White matter integrity in MDD + SUI, specifically in the corpus callosum and the anterior cingulate cortex compared to MDD and HCs. On network-based analysis, ↓ connections within subnetworks of the frontal lobe among MDD + SUI vs. HCs	Adults with MDD + SUI vs. MDD vs. HCs
Chen et al. (2021)	DTI	Significantly ↓ white matter compactness and integrity in the corpus callosum, cingulate gyrus, and caudate among MDD + SUIATT vs. both the depressed non-attempt and HCs	Adults with MDD + SUIATT vs. MDD vs. HCs

### 3.1. Functional MRI (fMRI)

#### 3.1.1. Task-based fMRI

The task-based fMRI research in STBs depends on the ability of a participant to complete a task in real time, and thus, may be a more proximally clinically important difference amongst patients. Task-based fMRI studies of the suicidal brain have focused on six primary tasks according to Dr. Van Heeringen: (1) Decision Making/Reward-Based Learning; (2) Emotional Pain and Affect Regulation; (3) Sensitivity to Social Stressors; (4) Cognitive Control/Response Inhibition; (5) Hopelessness; and (6) Impulsivity and Aggression. Unfortunately, within these 6 domains, only the first 4 have functional neuroimaging data consistent with our focused limitations.

##### 3.1.1.1. Decision making/reward-based learning

Examining the fMRI activation differences during a learning task which requires participants to maximize rewards earned through a sequence of lever pulls (the Three-Armed Bandit Task), Brown et al. found that while non-suicidal participants showed decreased impulsivity with increasing connectivity between the ventromedial prefrontal cortex (vmPFC) and parietal cortex, suicidal participants had *increasing* impulsivity with increased connectivity between these regions (16).

Suicide attempts have been found to be associated with greater activation in the right lateral orbitofrontal cortex (OFC) and decreased activation in the right superior frontal gyrus (SFG) while performing decision-making tasks in response to prototypical angry versus neutral faces (17).

In a recent meta-analyses that included work on decision making/reward-based learning, Li et al. concluded that brain activation in suicide attempters increased in the left insula but decreased in the bilateral fusiform gyrus compared to non-attempters across multiple learning-based fMRI tasks (18).

##### 3.1.1.2. Emotional pain, affect regulation

As a proximal and distal risk factor for suicide, social exclusion is of immediate importance to the suicidal patient. This is consistent with the concepts of “thwarted belongingness” in Joiner/Van Orden’s interpersonal theory of suicide, shining light on the vital importance of social support amongst persons at high risk for suicide.

Two fMRI studies (19, 20) have used the Cyberball task (where the participant is progressively excluded from a game) to examine social exclusion in suicidal populations. Both found a decreased activation in the anterior cingulate cortex (ACC) in their higher suicide risk groups. Specifically, Olie et al. found that suicide attempters displayed decreased activation in the left insula (as well as supramarginal gyrus) when compared to patients without any history of suicide attempt and healthy controls (19). Caceda et al. (20) found that the activation of the anterior insula during inclusion trials in suicide attempters was significantly decreased compared to depressed patients with and without suicidal ideation.

Emotion regulation is clinically relevant to multiple conditions, from self-injurious behavior to PTSD. Supportive of the clinical impression that emotion regulation is important in STBs, Miller et al. found that adolescents with suicidal ideation (SUI) showed increased activation in the dorsolateral prefrontal cortex (dlPFC) compared to healthy controls (21).

##### 3.1.1.3. Sensitivity to social stressors

Many studies have tried to examine the connection between the structures involved in affective processing and regions of interest in the suicidal brain. Clinically, this may be consistent with the concept of a cognitive distortion and related to such possible risks as low self-esteem, isolation, and unwillingness to seek help, though a comparison of the neurobiological changes associated with Cognitive Therapy is beyond the scope of this review.

In a study by Pan et al. participants made a gender selection for images of faces with or without affective valences (22). Consistent with the idea of a sensitivity to social stressors, adolescents with past suicide attempts showed an increased activation in ACC, dlPFC, sensory cortex, and temporal cortex during *angry* trials but a decreased activation in the same areas during neutral or happy face trials.

Ai et al. similarly utilized a gender discrimination task with individuals with past suicide attempts. Authors found that they had lower activation within the fusiform gyrus during emotional face processing across all stimulus types: happy, angry, sad, and scared (23).

Two major studies mixed resting state data with task-based functional tasks. Alarcon et al. found that depressed participants who had attempted suicide or had high suicidal ideation showed greater functional connectivity between the amygdala, dlPFC, dorsomedial prefrontal cortex (dmPFC), and precuneus compared to controls completing an emotional self-face recognition task where they considered if the faces looked like them, with valence varying between happy, sad, and neutral (24). Malhi et al. examined resting state functional connectivity (RSFC) in suicidal adults using an emotional face-word stroop paradigm in which congruent and incongruent images were flanked by the word “happy” or “sad.” During negative valenced incongruent trials, depressed participants (with and without suicidal thoughts and behaviors) had increased activity relative to healthy controls in the prefrontal cortex, frontopolar cortex, ACC, and posterior parietal cortex. At the same time, participants with STB’s showed increased activity among basal ganglia structures but decreased activity among the Medial Frontoparietal Network/Default Mode Network (M-FPN/DMN) structures with increasing suicide risk on measures, including the Columbia-Suicide Severity Rating Scale (C-SSRS) (25).

A recent machine learning study by Just et al. supported the involvement of structures important to all these processes among participants with suicidal ideation. When presented with affectively valenced verbal stimuli, specifically the words death, cruelty, trouble, carefree, good, and praise, group differences in activation in the medial superior frontal cortex, inferior parietal, medial temporal, ACC, and inferior frontal gyrus (IFG) were found (26).

##### 3.1.1.4. Cognitive control/response inhibition

Impairments in top-down cognitive control and response inhibition have clear clinical relevance to suicide but have been studied very little in task-based fMRI research in this population. However, Richard-Devantoy et al. used a Go-No-Go task and found that deficits in cognitive inhibition (in relation to the IFG, thalamus, OFC, and parietal cortex) were related to the depressive, but not specifically, STB vulnerability risk (27). In contrast, the meta-analysis involving neuroimaging studies using the monetary incentive delay task and the stop signal task with over 5,000 participants aged 9–11 could not delineate between those with suicidal ideation from those with suicidal behaviors (28). Although our belief is that there is a difference in this domain between those with suicidal ideation and those who attempt, evidence is scant and will depend on future work of our own and others.

#### 3.1.2. Resting-state fMRI

While performance on fMRI tasks and task-based connectivity studies can show abnormal/atypical recruitment of structures theorized to be essential in relevant cognitive tasks, resting-state fMRI (rsfMRI) studies allow a view of default self-referential thought processes while the participant is not engaged in a specific task. It, therefore, is used to analyze which relevant systems have robust or weakened ‘default’ connections or communications (29–31) within and between networks, defined as the level of increased, decreased, or mixed functional connectivity between them, or RSFC. Each defined network is organized based on theoretical common functions. In the interest of clarity, we will use Uddin et al.’s (32) definitions of major networks as a reference for the resting-state studies in our review, as they clarify the involved neuroanatomy and are analogous to established networks familiar in the research domain.

Uddin proposes the following six main networks and their primary functions: (1) the Lateral Fronto-Parietal Network/Cognitive Control Network (**L-FPN/CCN**) = *functions include executive functions, such as goal-oriented cognition, working-memory, inhibition, and task switching*; (2) the Pericentral/Somatomotor Network (**PN**/Somatomotor) = *functions include involvement in motor processes and somatosensory processing*; (3) Occipital Network/Visual Network (ON/VN) = *functions include visual processing*; (4) Dorsal Frontoparietal Network/Attention Network (**D-FPN**/**AN**) = *plays a broad role in visuospatial attention. The functions of this system are to prepare and apply top-down selection for stimuli and responses*; (5) The Midcingulo-insular Network/Salience Network (**M-CIN/SN**) = *has a broad role in identifying important, or salient, information. Salience processing involves the detection of behaviorally-relevant environmental stimuli and may include internally-generated (i.e., remembered) information*; and the (6) **M-FPN/DMN** = *functions likely involve formation, temporal binding, and dynamic reconfiguration of associative representations based on current goal-states, detecting the associative relevance of internal and external stimuli, and providing value coding and elaboration to perceived events. Other accounts suggest M-FPN function accommodates predictive coding, semantic associations, and plays a role continuously monitoring the environment*.

Though abnormal RSFC can be complicated, ranging from nodal (i.e., region to region) to whole brain (as it sounds) analysis and involving established networks or networks defined within each individual study, it may become increasingly relevant to clinical practice. One limitation may be the inherent assumption of the specific functions of each interconnected area. We present RSFC studies grouped according to main findings. The most consistent findings among suicidal participants seem to occur between the M-FPN (default network/DMN) and the M-CIN (salience network/SN). Many studies showed decreased RSFC between M-FPN/DMN and M-CIN/SN. When we talk about connections between networks, we clearly mean connections between the regions or nodes that comprise networks. In other words, resting state or intrinsic brain networks in fMRI are best thought of as a collection of regions that show correlations in terms of their fluctuating activity.

Qiu et al. (33) looked at depressed participants with a history of suicide attempts compared to those without and found that in both groups, as suicidal ideation increased, functional connectivity decreased between the pregenual anterior cingulate cortex (pgACC part of M-CIN/SN) and the superior frontal gyrus (M-FPN/DMN). Among depressed suicidal adults, Du et al. (34) found that the SUI group exhibited *decreased* functional connectivity between the right ACC (M-CIN/SN), the orbito-medial prefrontal cortex (M-FPN/DMN), and the right middle temporal pole (within the D-FPN/AN) compared to non-suicidal depressed and control groups. Yang et al. (35) found that between depressed participants with past attempts and those without attempts, those with past attempts showed decreased RSFC (decreased positive correlation) in the right inferior front orbital gyrus (within the M-FPN/DMN) to the left inferior parietal lobule (within the M-CIN/SN). They also found that compared with non-attempters, those with past attempts had decreased gray matter volume (GMV) in the right inferior frontal orbital gyrus (IFOG) and left caudate (CAU) but increased GMV in the left calcarine fissure.

In a study by Stange et al. (36), suicidal participants showed decreased connectivity between the right middle frontal gyrus/SFG (L-FPN/CCN) and the M-FPN/DMN and decreased connectivity between the precuneus (L-FPN/CCN or M-FPN/DMN) and the Salience Network (SN). The difference in RSFC within areas of M-FPN/DMN was greater than that between M-FPN/DMN to M-CIN or to L-FPN/CCN.

Cao et al. (37) analyzed low frequency RSFC and found participants with STB’s had increased connectivity in the right superior temporal gyrus (STG) (M-FPN/DMN), left MTG (M-FPN/DMN), and left middle occipital gyrus (ON/VN) but decreased connectivity in the left SFG (M-FPN/DMN) and left MFG (M-FPN/DMN), at least compared to non-suicidal clinical participants. Another study by this group (38) found that participants with past attempts had decreased RSFC between the left MFG (L-FPN/CCN) and the left SPG (D-FPN/AN) compared to the non-attempt group and decreased RSFC between the left superior frontal gyrus (M-FPN/DMN) and the right ACC (M-CIN/SN).

Several studies showed an increase in connectivity between the M-FPN/DMN and M-CIN/SN. Zhang et al. (39) specifically focused on RSFC between the bilateral amygdala and whole-brain activation and found increased connectivity between the right amygdala (M-CIN/SN) and bilateral paracentral lobule/precuneus (part of M-FPN/DMN) in a suicidal behavior (suicidal ideation and/or past suicide attempt) group relative to non-suicidal and healthy-control groups.

Kang et al. (40) found mixed results, with suicide attempters displaying significantly increased functional connectivity of the left amygdala (within the M-CIN/SN) with the right insula (within the M-CIN/SN) and the left superior orbitofrontal area (within the DMN) and increased functional connectivity of the right amygdala (within the M-CIN/SN) with the left middle temporal area (within the D-FPN/Attn). Wei et al.’s (41) study similarly found that suicidal patients with depression showed greater amygdala (within the M-CIN/SN) to precuneus/cuneus (within the M-FPN/DMN) RSFC compared with non-suicidal patients and healthy controls.

Cao et al. (42) showed that a suicidal (history of an attempt) depressed group demonstrated decreased RSFC connectivity between the anterior M-FPN/DMN and left L-FPN/CCN but *increased* connectivity between the L-FPN/CCN and M-CIN/SN.

The next most common findings were abnormal RSFCs between the M-FPN/DMN and L-FPN (CCN), adding to the findings of Stange, Cao and others.

An RSFC analysis of treatment changes by Shu et al. (43) showed that prior to treatment, participants showed increased baseline activity in the left posterior cerebellar lobe, right ACC (within the M-FPN/DMN), left caudate (within the L-FPN/CCN) nucleus, and left superior frontal cortex (within the M-CIN/SN). After combined treatment, patients showed increased activity in the left middle occipital cortex and left precuneus (within the L-FPN/CCN).

After this, intra-network abnormalities show up in multiple studies, focusing on the M-FPN/DMN.

Zhang et al. (44) used an independent component analysis to show that RSFC within the M-FPN/DMN was increased in the left cerebellum but decreased in the posterior cingulate cortex (PCC) and right precuneus among suicidal (SUI & STBs) versus healthy controls. Network analysis by Chen et al. (45) found increased connectivity between the frontal (M-FPN/DMN) and parietal lobes in comparison to the healthy controls.

Yang’s work (35), mentioned above, also found participants with past attempts had increased RSFC between the right IFOG and left rectus gyrus (both in M-FPN/DMN).

In a study examining trauma and suicide in adults with PTSD, increased functional connectivity between reward and control regions (primarily under the M-FPN/DMN of Uddin’s definition) was found to be positively correlated with suicidality (46).

Lee et al. (47) found that suicidal patients (past attempts) with depression had significantly increased RSFC in tracts from an anteriorly defined division of the right parahippocampal gyrus (within the M-FPN/DMN) to a posteriorly defined division of the left parahippocampus.

Schreiner et al. (48) examined suicidal adolescents and found more evidence for involvement of the precuneus/cuneus (within the M-FPN/DMN) in the suicidal mind. They showed that in suicidal participants, as suicidality increased, RSFC increased between the right precuneus (M-FPN/DMN), right IFG (M-FPN/DMN), and cerebellum; and between the left PCC (M-FPN/DMN), left cerebellum, and cingulate gyrus.

Using whole brain analysis, Kim et al. (49) proposed a subnetwork of decreased RSFC among participants with suicidal ideation consisting of the “orbitofrontal cortex (within the M-FPN/DMN), especially the left SFT (M-FPN/DMN), pars orbitalis, left MFG, and right olfactory cortex.”

Most promising may be the recent attempts to apply machine learning to one or more MRI modalities. Gosnell et al. (50) distilled five prominent RSFC patterns amongst participants with suicidal ideation or past attempts, including: (1) decreased RSFC between the rSFG (M-FPN/DMN) and insula (M-CIN/SN); (2) increased RSFC between the left habenula (possibly M-CIN/SN) and right parahippocampus (M-FPN/DMN); (3) increased connectivity between the left frontal middle orbital gyrus (M-FPN/DMN) and left rolandic operculum (L-FPN/CCN); (4) increased connectivity between the left putamen (within the FPN) and the cerebellar vermis; and (5) decreased connectivity between the amygdala (within the SN) and middle temporal pole (within the D-FPN/AN).

Dai et al. (51) used ICA (machine learning) to conclude that the relevant structures were the right inferior temporal gyrus (within the L-FPN/CCN), left IFG (within the DMN), right angular gyrus (within the L-FPN/CCN), left inferior parietal cortex (IPC) (within the L-FPN/CCN), left rolandic operculum (within the L-FPN/CCN), and right dorsolateral superior frontal gyrus (within the DMN-M-FPN).

Suicide attempt-related altered RSFC was also observed in a Graph Analytics analysis (machine learning) by Stumps et al. (52) within the M-CIN(SN), cognitive-control (L-FPN), M-FPN, and visual networks.

There were a variety of studies showing abnormal connections between other networks, including the D-FPN/Attention Network, PN/Somatomotor Network, and ON/Visual Network.

In line with the research of Du and Kang above, Chase et al. (53) found that patients with SUI (but not necessarily historical attempts) had increased connectivity between the dorsal PCC(M-FPN/DMN) and MTG (D-FPN/Attn).

Serafini et al. (54) in a review of RSFC work showed a mixed increased connectivity/decreased connectivity pattern among networks but could not distinguish findings between pathologies.

Along with abnormalities in the M-FPN/DMN above, Lee also found increased RSFC in tracks from the temporooccipital part of the right inferior temporal gyrus (within the L-FPN/CCN) to the frontal eye fields of the Dorsal Attention Network (i.e., within the D-FPN). They also found decreased RSFC between the medial frontal cortex (within the M-FPN/DMN) and the right supplementary motor cortex (within the PN/Somatomotor network).

### 3.2. Brain morphometry

Another way to examine the suicidal brain is through morphometric analysis. Here, we try to make it more directly accessible to the clinician. This modality measures the physical makeup of brain structures by examining measures such as cortical thickness, cortical surface area, and/or cortical volume of the relevant brain areas. The implication is that the neural systems which may be hypo/hyperactive during functional processing may have altered physical attributes. Many morphometric studies have been done to find alterations in those with STBs.

The current review identified such studies which reported associations between these morphometric measures and suicidal behavior.

One of the earlier studies addressing the association between history of suicidal attempts in elderly, depressed individuals and both cortical and sub-cortical abnormalities was attempted by Hwang et al. (55). In that study, authors used voxel-based analysis and were able to show widespread gray matter volume (GMV) reduction in the frontal (i.e., left medial, bilateral superior, right middle, right inferior, and left posterior frontal cortices), parietal (i.e., left superior, right inferior, and left lateral parietal cortices), occipital gyrus (i.e., left cuneus), left STG, and sub-cortical (i.e., bilateral insula, left lentiform nucleus, right claustrum, bilateral midbrain, bilateral culmen, and right inferior and bilateral superior semilunar lobules) brain regions in late-onset geriatric depressed individuals with a history of suicide attempts compared to those without previous attempts. Here, volume reduction was most prominent within the dmPFC, consistent with impairment in reward-based learning and top-down executive control.

Wagner et al. reported cortical thinning in the left dorsolateral, ventrolateral prefrontal, and ACC in depressed adults with documented suicidal behavior, i.e., high-risk group of suicide as compared to depressed adults with a non-high risk for suicide (56).

A study by Huber et al. specifically found white-matter volume differences in the left ACC between veterans with a history of attempting suicide relative to veterans with a history of SUI (57). Along with RSFC data previously mentioned, the work by Barredo and colleagues found that cortical thickness of the ACC/PCC was shown to predict the functional connectivity between the lateral pars orbitalis and anterior cingulate/paracingulate control regions (46).

Wang et al. specifically reported significant differences in GMV in the bilateral MFG across patients with mood disorders and suicidal behavior, patients with mood disorders without suicidal behavior, and healthy controls (58). However, they did not find significant differences between participants with SUI and those with a history of actual suicide attempts.

Ding et al. used a region-specific approach to study differences between suicide attempters with a past history of mood disorders and suicidal behavior, participants with a mood disorder but not suicidal behavior, and healthy controls with neither (59). Reduced cortical volume was observed within the left ventrolateral prefrontal cortex in suicide attempters compared to both control groups. In addition, the orbitofrontal and dorsal prefrontal cortices (but not medial prefrontal cortex) also showed reduced cortical measures in suicide attempters compared to healthy controls. This is consistent with task-based and resting-state fMRI studies showing generally both reduced top-down executive control and impairment in reward-based learning regions and networks. Structurally, there were significant GMV decreases among suicide attempters across clinical conditions compared to non-attempters. Diffusion Tensor Imaging (DTI) findings also showed significantly reduced fractional anisotropy among those with past attempts versus those without (60). In a study of future suicide attempters (i.e., individuals attempting suicide between baseline and follow-up assessment) with mood disorders, participants showed lower baseline ventral and rostral prefrontal GMV compared to those who did not attempt (61). Besides the studies examining participants with depression and/or previous suicide attempts, distinct markers that included an involvement of frontal regions, particularly reduced cortical volume within the left MFG and cortical thinning within the posterior frontal lobe including the bilateral precentral gyrus, were also found in individuals with current SUI as compared to healthy controls without even a family history of psychiatric disorders or suicide attempts (62). Interestingly, greater cortical surface area and cortical volume within the left dorsolateral prefrontal gyrus were reported to be associated with reduced SUI in a non-clinical population with mild levels of stress and perceived lack of social support (63). This would be consistent with increasing behavioral control and top-down influence on impulsivity with decreasing pathology.

In a recent study by Yang et al. authors found that suicidal depressed patients had reduced GMV in the right IFG and left caudate but increased GMV in the left calcarine fissure (35), areas associated with visual processing not irrelevant to potential affective or social stimuli.

A recent study by Kang et al. involving individuals with depression with and without a history of suicide attempts showed altered morphometry in the lateral parietal and occipital brain regions along with frontal areas. In that study, depressed patients with past suicide attempts were found to have larger surface area within the left postcentral and left lateral occipital areas and large cortical volume within the left postcentral and left lateral orbitofrontal areas, whereas smaller surface area within the left SFG was found (64). In another recent study by Harenski et al. criminal offenders with a history of suicide attempts had widespread decreased gray matter within both cortical and sub-cortical regions, including the PCC/precuneus, IPC, dorsal prefrontal cortex, amygdala, insula, superior occipital gyrus, cuneus, and cerebellum (65). Using local shape volume analysis, researchers specifically found significant volumetric differences between suicidal and non-suicidal depressed individuals in the left amygdala, left hippocampus, left putamen, bilateral pallidum, and bilateral thalamus (66). In another study however, it was only the reduced volume of the right hippocampus that was most prominent in participants with a recent history of suicide attempts within the past 2 months as compared to healthy individuals (67). This may represent limited or impaired processing of new information relative to old beliefs and memories, consistent with Van Heeringen’s framework. Interestingly, in a postmortem study, compared to healthy controls, the suicidal depressed participants had an increased neuron number in CA2/3 subregions of the hippocampus gyrus (68).

In a study of suicide attempters with a family history of suicide and personal history of violent suicide attempts, Jollant et al. found an association between family history of suicide and reduced volume within the bilateral temporal regions, right dlPFC, and left putamen, as well as between violent methods of attempt and increased bilateral caudate and left putamen volumes (69). Reduced GMV in the dorsal striatal structures, particularly bilateral putamen and caudate, were associated with greater implicit SUI observed from suicide-related outcomes from the death version of the Implicit Association Test (IAT) (70, 71).

Several studies also reported cortical alterations within the temporal and parietal lobes but not the frontal lobe.

Reduced GMV within the right STG was observed in adolescents with MDD and a history of suicide attempts compared to adolescents with MDD but without any history of suicide attempts (72), whereas care-giver reported STBs were also associated with decreased volume at the left bank of the superior temporal sulcus in children (73). In another study on adolescents, McLellan et al. reported reduced volume of the right STG in adolescents with treatment-resistant depression and a history of suicide attempts as compared to healthy adolescents (74). Compared to healthy controls, patients with MDD and a history of suicide attempts in Peng et al.’s work showed decreased GMV within the right MTG and increased GMV within the right parietal lobe (75). Authors reported decreased GMV in the left limbic cingulate gyrus for the depressed suicidal group compared to the depressed non-suicidal group. Somewhat contrary to Peng’s work, patients with an attempt history have also been shown to have decreased GMV in the left anterolateral region of the parietal lobe as well as in the right cerebellum (76).

A recent study by the ENIGMA-MDD working group of over 18,925 participants examined morphometric differences between healthy controls, depressed participants, and participants with a history of attempted suicide and found multiple differences between the groups (77). Regarding volumetric differences, the thalamus and right pallidum were significantly smaller in depressed attempters compared to depressed and healthy controls. Regarding cortical surface area, depressed attempters had smaller cortical surface area of the left cuneus, left inferior parietal, left rostral middle frontal, and right pericalcarine cortex compared with healthy controls, but only the inferior parietal cortex was clinically distinct from depressed clinical controls. Lastly, in terms of cortical thickness, although there was not a significant difference between depressed attempters and clinical controls, attempters did display significantly lower cortical thickness in the left rostral middle frontal region. The authors concluded that these findings suggested impairment in decision making, impulsivity, and planning as well as attention and the concept of self (77). However, please note that a significant difference in such large studies does not mean the effect size is clinically meaningful. In other words, a clinically insignificant volumetric difference could be statistically significant due to the large number of subjects studied. For example, in this study, for the left pallidum and right nucleus accumbens subcortical volumes, the difference between clinical and healthy controls did not reach statistical significance after correction for multiple comparisons. Therefore, statistically significant differences may not necessarily translate to clinical significance, but it can inform next steps and build a future-focused plan for translational researchers.

Most recently, Sarkinaite et al. (78) published findings that showed volumetric differences between participants with past suicide attempts and healthy controls in the frontal and temporal cortex thickness and volume of the hippocampus. Notably, with number of attempts as a covariate, participants with increasing number of past suicide attempts showed decreasing thickness of temporal cortex in the inferior middle and temporal cortex.

**Figure 1:**
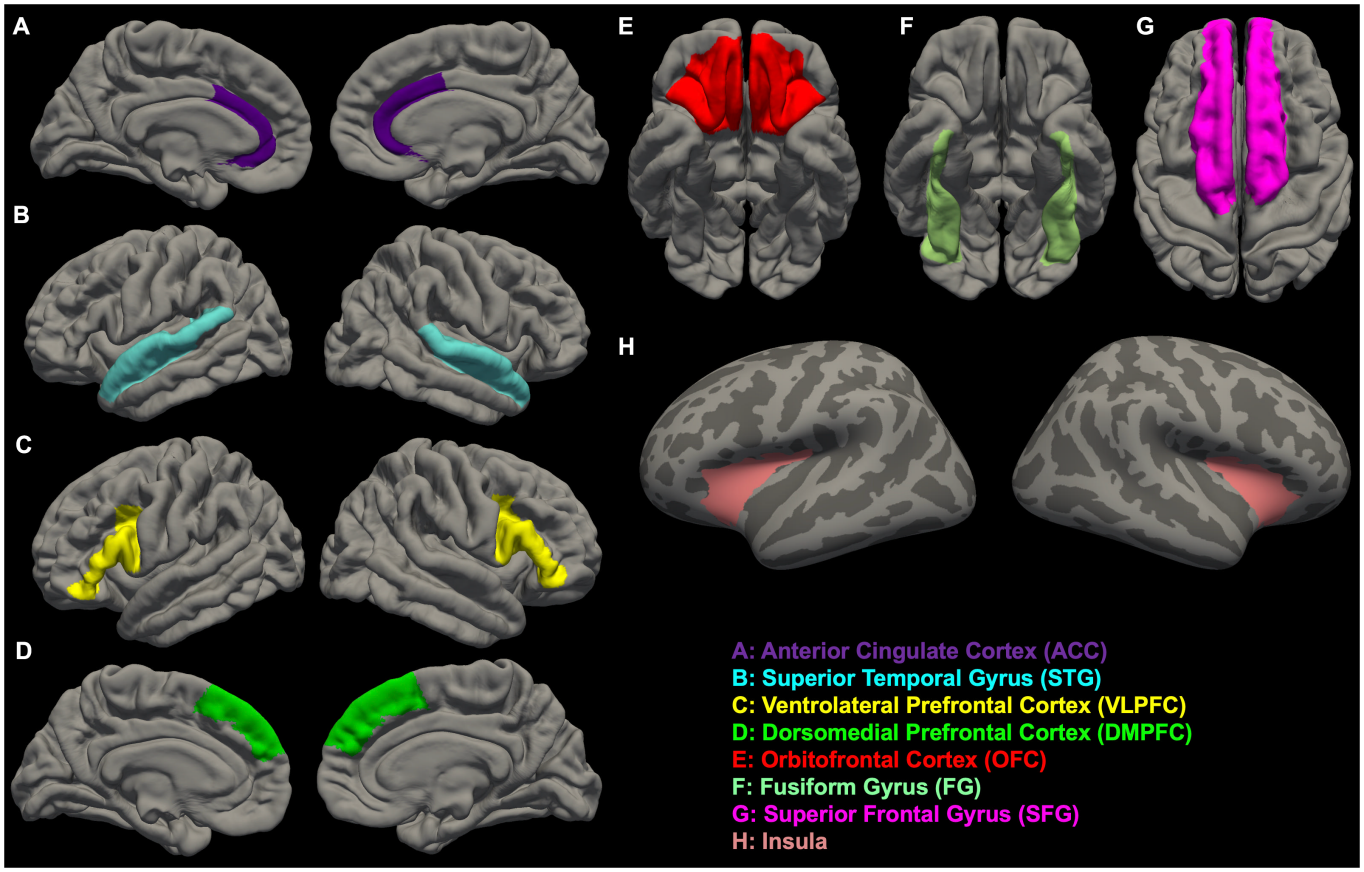
Overview of brain regions **(A–H)** that are most commonly found to be involved in functional MRI and brain morphometry research of suicidal thoughts and behavior.

**Figure 2:**
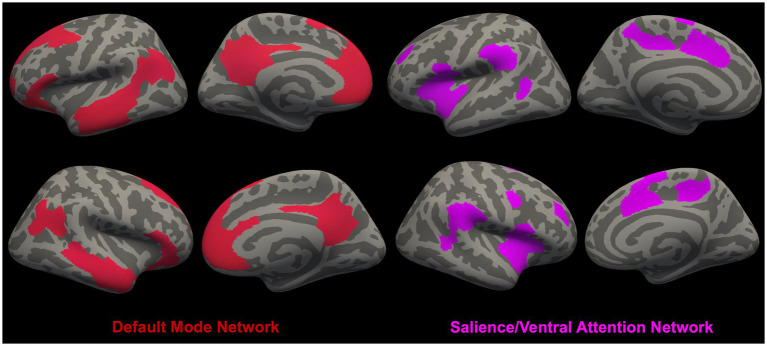
Overview of brain networks [i.e., Default Mode Network (DMN) (79) and Salience/Ventral Attention Network (SN/VAN) (80)] (81) that are most commonly found to be involved in functional MRI and brain morphometry research of suicidal thoughts and behavior.

In Figures 1, 2, we provide an overview of brain regions (Figure 1) and networks (Figure 2) that are most commonly found to be involved in functional MRI and brain morphometry research of suicidal thoughts and behavior. Both the figures are generated through FreeSurfer 7.3.2 (82, 83) and Yeo’s 17-network atlas (84).

### 3.3. Diffusion-weighted MRI

The fourth neurobiological modality that we included in this study was Diffusion Tensor Imaging (DTI). This method analyzes the robustness of water diffusion within white-matter structures of the brain to find associated differences between patient populations and healthy controls or other cohorts. DTI also examines the physical “highways” within and between significant structures. While fMRI gives information on “in the moment” electrical communication that could conceivably indicate functional robustness of structures and pathways, DTI is a direct measure of the physical robustness of those structures and pathways. To that end, it may be considered as a more concrete/persistent measure to estimate the difference between healthy controls and the suicidal patients. Again, most of the modalities can be accomplished in a relatively short scanning session and are more potentially accessible to the working clinician.

Recent advancements in imaging have allowed the scientists to study differences in white-matter integrity, compactness, or structural connectivity in clinical populations – the “quality of the highways” so to speak. These studies use parameters such as apparent diffusion coefficient (ADC-speed of water flow regardless of direction), fractional anisotropy (FA-diffusion of water molecules in a particular direction), and edge-weight (white-matter structural connectivity) to measure connections between regions of interest (85, 86). Edge weight has been considered a potentially more appropriate parameter to measure the strength of structural connectivity because it takes into account both the number of white-matter fibers and the size of the regions of interest (87). Our focused review identified studies which reported associations between these measures and suicidal behavior.

In a study of young adult healthy controls and young adults with MDD with and without a history of suicide attempts conducted by Jia et al. reduced FA was found in the (a) left anterior limb of the internal capsule (ALIC) for suicide attempters compared to non-attempters and healthy controls, (b) right frontal lobe (subgyral) for suicide attempters compared to healthy controls, and (c) right lentiform nucleus (putamen) for suicide attempters compared to non-attempters (88). In another similar study by Jia et al. it was also found that compared to healthy controls, depressed suicide attempters had significantly lower mean percentage of fibers projecting from the ALIC to the left medial frontal cortex, left OFC, and left thalamus. Compared to depressed non-suicide attempters, depressed suicide attempters had significantly lower mean percentage of fibers projecting from the ALIC to the left OFC and left thalamus (89). However, in a study involving panic disorder and suicide attempt, several regions, including the retrolenticular part of the internal capsule, splenium of the corpus callosum, superior and posterior corona radiata, posterior thalamic radiations, sagittal stratum (including the inferior longitudinal fasciculus and inferior fronto-occipital fasciculus), and superior longitudinal fasciculus, showed increased FA in individuals with panic disorder and history of suicidal attempt (PD + SA) compared to individuals with panic disorder but without any history of suicidal attempt (90). For the PD + SA group, Kim et al. also found that for two regions (i.e., right retrolenticular part of the internal capsule and bilateral posterior thalamic radiations), there was a significant positive association between FA and SUI.

In another study, lower baseline FA was found in the left ALIC, bilateral dmPFC, and right dorsal cingulum for future suicide attempters (i.e., who attempted suicide between the baseline and follow-up assessment) compared to non-attempters (61). In that study, compared to the non-future suicide attempt group, both future suicide attempters with or without a history of suicide attempts had lower FA for the left dmPFC, right dlPFC, and left ALIC. Future suicide attempters with a history of suicide attempts also showed lower FA in the right dmPFC and right dorsal cingulum. Authors found that after an exclusion of four participants with alcohol/substance use disorder, the left ventral prefrontal cortex also had lower FA for the future suicide attempters relative to non-attempters.

Fan et al. also found lower FA in the dorsal and ventral frontal regions that included the uncinate fasciculus for individuals with MDD and a history of suicide attempts as compared to non-suicide attempters (60).

In terms of structural connectivity parameters, it was determined that compared to individuals with MDD without SUI, individuals with MDD and SUI had reduced structural connectivity/edge weights across cortical (i.e., rostral middle frontal cortex, superior parietal cortex), subdivisions of the inferior frontal cortex (i.e., pars triangularis and pars orbitalis, frontal pole, and lateral occipital cortex), and sub-cortical (i.e., pallidum, thalamus, putamen, and caudate) regions in the left hemisphere (91). In terms of FA also, the frontal areas, especially right dmPFC and white-matter bundles in several regions, including the bilateral inferior fronto-occipital fasciculus, bilateral uncinate fasciculus, body of corpus callosum, right anterior limb of internal capsule, right external capsule, left posterior thalamic radiation, and bilateral posterior corona radiata, showed lower FA amongst suicide attempters with MDD compared to non-attempters with MDD and healthy controls (92, 93).

Another structural connectivity study showed that compared to euthymic non-attempters and healthy controls, there was significantly decreased structural connectivity in euthymic suicide attempters in the connections between the left olfactory cortex and left anterior cingulate gyrus, as well as a clear trend of decreased connectivity between the right medial orbital, SFG, and the right rectal gyrus and between the right calcarine fissure and both the left superior and middle occipital gyrus (94). Hwang et al. reported greater edge weight in the left PCC and greater structural connectivity strength of local connections amongst participants who were military veterans with prior suicide attempts in comparison to those with SUI only and with no suicidal behavior (95).

A 2021 study by Chen et al. evaluated white-matter integrity (generalized fractional anisotropy) and white-matter compactness (normalized quantitative anisotropy) among depressive patients with and without past suicide attempts (96). On a voxel-based (region of interest) analysis, participants with past suicide attempts had significantly lower white matter compactness and integrity in the corpus callosum, cingulate gyrus, and caudate than both the depressed non-attempt and the healthy control groups, with differences between the attempt group and healthy control group reaching statistical significance (96).

The same researchers evaluated white matter density and integrity among depressed patients with suicidal ideation but no history of attempts (97). In the voxel-based analysis, white matter integrity was found to be decreased in the suicidal ideation group, specifically in the corpus callosum and the ACC compared to depressed, non-SI, and healthy control participants. On RSFC, the suicidal ideation group had weaker connections within subnetworks of the frontal lobe compared to healthy controls but did not find differences between suicidal ideation and depression in suicidal participants.

## 4. Discussion

The concept of maladaptive thinking in depression is well understood by most clinicians (98). The notion that patients may develop maladaptive thinking in/or about social interactions is likely not surprising. However, to grasp the comprehensive picture of what is happening neurobiologically, providers must look to the evolving literature in neuroscience and neurobiology. Among our sample we have found many indications that functional, structural, RSFC, and diffusion-weighted MRI studies are beginning to bridge this translational gap well.

Task based fMRI studies show abnormal activation in prefrontal, subcortical, and limbic regions. Specifically, areas important for emotional processing, reward-based learning (value estimation), emotional regulation/social exclusion, relative representation/sensitivity to affective stimuli, and cognitive control/response inhibition show abnormal activation, though in some cases (18), they contradict other studies. It may be that the same regions (e.g., left insula) are increasingly active in some cognitive challenges but less activated than controls in others, but this would not be contradictory to Van Heeringen’s model. Complicating the current growing body of research is the comparison of suicidal individuals with past attempts, versus those without, versus those with ideation and those without. At this stage it may simply be important to keep the focus on what regions and processes are relevant as data grows and models continue to develop.

Among our sample, rsfMRI enriches the functional data by showing the major networks with abnormal connectivity among patients with MDD and STBs. From machine learning to strictly RSFC studies, the most relevant networks are clearly the M-FPN/DMN and M-CIN/SN and their communication within and between each other and with top-down control areas of the L-FPN/CCN. With the vast and complex roles of the M-FPN/DMN and M-CIN/SN, it is clear that even in the resting state, areas important for learning, affective and social processing, and cognitive control are affected, but this research also lends depth to the increased weight of an abnormally functioning “salience judge” at the cost of new information coming in from all sides. The mix of participants (adults, adults and adolescents, older adults, combat veterans, and convicted criminals) and type of STB being studied (standard scale score, suicide attempt, or suicidal ideation) of course complicates these already complicated findings, but data continues to grow.

Helping to enlighten a picture of abnormal communication amongst regions and networks, DTI data among our sample similarly supported differences in major tracts such as the ALIC and uncinate fasciculus (UF), important in communicating between structures of the reward-based learning network, along with differences in edge weight and FA among and between structures important for emotional processing, reward-based learning (value estimation), emotional regulation/social exclusion, relative representation/sensitivity to affective stimuli, and cognitive control/response inhibition. The clear difference among our DTI sample and the other methodologies, however, is that among participants with MDD and STBs, all of these measures were found to be decreased in comparison to control groups.

This is further supported in our sample by the morphometric studies generally showing that structures involved in both top-down and bottom-up emotional processing, visual and language processing, impulse control, and affective processing are atypical across the board. Decreased GMV/thickness/area were found in frontal systems such as the IFG and OFC, ventral-lateral prefrontal cortex, dlPFC and temporal regions, and in suicidal individuals, there are clear reductions in GMV in subcortical (putamen and caudate) and limbic (hippocampus-MFPN/DMN) areas as well. Intuitively, these measured differences represent more long-term changes among the relevant structures and networks that a patient would depend on as stressors and, hence, the risk of STBs accumulate.

Previous literature reviews are generally consistent with our results, though many have incorporated different samples with different conditions that inhibit direct comparison. Especially relevant to our review are the work of Jollant (5), Desmyter (99), Zhang (100), Martin (101), Schmaal (10), and Auerbach (11), among others (102–106). Studies over the last 20 years have increasingly showed a relation between emotional pain and physical pain (107–109). Work by Olie et al. has specifically examined the increasing relevance of social exclusion to affective pain and suicide and further discussed the association between neuroimaging findings of social exclusion and suicide risk (110). They found that while the normal response to the affective pain of social exclusion increases activity in the anterior insula, ACC, and inferior OFC in normal controls, suicidal individuals show a decreased activation in these same regions, even compared with non-suicidal patients with a mood disorder.

In short, we are not proposing a grand new theory that is all encompassing, for that would take many more variables into account, which have their own emerging literature, such as genetic, socioeconomic, and cultural factors. Also biochemical, MEG, SPECT, and PET scanning and incorporating the rich and ever evolving psychometric data would also need to be considered. We are simply proposing a framework to begin applying emerging neurobiological data in a clinical and chronological way in conjunction with already used measures, such as psychometrics and clinical assessments, so that as a patient encounters various diatheses and stressors, their clinician will be able to look to this framework to address a complex problem in comprehensive but clinically feasible way. With genuine and earnest collaboration in translational medicine, imaging, and neuroscience, combined with machine learning and worldwide research consortiums focusing on suicidal thoughts and behaviors, and replication of findings, especially in these diverse and complicated modalities, collaboration can shrink the time from new discoveries to clinical intervention. There are few things more urgent than attempting it.

Our simple framework is this: First, morphometric changes may be more observable early on from genetic and environmental stressors but also long standing atypical cognitive processing. Second, abnormalities in diffusion-weighted projections will become apparent, implying increasingly longstanding atypical networks and relevant ROI communication, and demonstrable on scanning. Third, abnormalities in processing new information, especially negative social and affective valenced-relevant stimuli tethered to language and facial processing, will be demonstrated on rsfMRI/RSFC analysis, as evident by increased communication between networks (M-CIN/SN to L-FPN/CCN), implying maladaptive rumination of faulty negative information and decreased communication between new, contradicting affective/relevant processing areas and value estimation/strategy adjustment networks (M-CIN/SN TO M-FPN/DMN). Fourth, the gap between value and risk estimation will widen on fMRI tasks immediately prior to the STBs.

Ultimately, we hope to start to construct a chronological framework of early diatheses, developing stressors, whether distal or proximal, and correlating their neurobiological fingerprint across MRI modalities and behavioral task performance. We will do this through continued task/theory-based fMRI and network studies, structural/morphometric, RSFC, and DTI research. We will continue to develop machine learning evaluations through each modality and across them first using classification and machine learning techniques to quickly determine biosignatures that directly affect suicide risk and improve our model. We will then use a regression analysis to analyze level of risk and sequence mining to predict proximal neurobiological changes. Through this comprehensive and accelerated approach, we hope to begin to capture a clinically relevant and useful point-of-care tool that can accurately and thoroughly assess risk of suicidal ideation and attempt. Then, with extensive collaboration, those in the field of neurobiological suicide research can shift into evaluating the most effective interventions at each specific time that will prevent it. Lastly, given that functional connectivity studies and diffusion tensor imaging have a particular drawback: neither are in a position to assess directed functional or effective connectivity (111). In other words, one gets a single number for the connectivity between two regions – as opposed to separate estimates of the directed influence of one region on another, and the reciprocal influence. This is important when talking about the distinction between bottom-up and top-down processes in functional brain hierarchies. In consequence, given our expertise in cutting-edge directed functional (e.g., Granger causality) and effective (e.g., dynamic causal modeling) brain connectivity techniques (29–31, 112–117), we will aim to see how analyses of directional connectivity nuance the emerging picture of suicidal thoughts and behavior described above. Emerging research in directed functional and effective connectivity will surely prove invaluable.

## Author contributions

MD conceived the presented idea, performed the literature search and wrote the initial draft. KB substantially contributed to interpreting the relevant literature and writing the manuscript. EC performed the literature search and contributed to writing of the manuscript. JB substantially contributed to the conception, interpretation, and writing of the manuscript. AD contributed to the writing of the manuscript and edited various versions of the draft. SB conceived the presented idea, performed literature search, wrote the initial draft, and supervised all aspects of the study. All authors contributed to the article and approved the submitted version.

## Funding

This work was funded by GRT-00092 at Boys Town National Research Hospital.

## Conflict of interest

The authors declare that the research was conducted in the absence of any commercial or financial relationships that could be construed as a potential conflict of interest.

## Publisher’s note

All claims expressed in this article are solely those of the authors and do not necessarily represent those of their affiliated organizations, or those of the publisher, the editors and the reviewers. Any product that may be evaluated in this article, or claim that may be made by its manufacturer, is not guaranteed or endorsed by the publisher.

## References

[1] World Health Organization. Preventing suicide: a global imperative. Geneva, Switzerland:WHO Press (2014). Available at: https://apps.who.int/iris/bitstream/handle/10665/131056/9789241564878_eng.pdf

[2] Curtin SC Heron M. Death rates due to suicide and homicide among persons aged 10-24: United States, 2000-2017. NCHS Data Brief. Washington, DC, United States: U.S. Department of Health and Human Services (2019) 352:1–8. Available at: https://pubmed.ncbi.nlm.nih.gov/31751202/31751202

[3] Centers of Disease Control and Prevention. Leading causes of death and injury. (2023).

[4] Posner K Brown GK Stanley B Brent DA Yershova KV Oquendo MA. The Columbia-suicide severity rating scale: initial validity and internal consistency findings from three multisite studies with adolescents and adults. Am J Psychiatr. (2011) 168:1266–77. doi: 10.1176/appi.ajp.2011.10111704, PMID: 22193671 PMC3893686

[5] Jollant F Lawrence NL Olié E Guillaume S Courtet P. The suicidal mind and brain: a review of neuropsychological and neuroimaging studies. World J Biol Psychiatry. (2011) 12:319–39. doi: 10.3109/15622975.2011.556200, PMID: 21385016

[6] van Heeringen K. The neuroscience of suicidal behavior. Cambridge, England: Cambridge University Press (2018).

[7] Van Orden KA Witte TK Cukrowicz KC Braithwaite SR Selby EA Joiner TEJ. The interpersonal theory of suicide. Psychol Rev. (2010) 117:575–600. doi: 10.1037/a0018697, PMID: 20438238 PMC3130348

[8] McGirr A Turecki G. The relationship of impulsive aggressiveness to suicidality and other depression-linked behaviors. Curr Psychiatry Rep. (2007) 9:460–6. doi: 10.1007/s11920-007-0062-2, PMID: 18221625

[9] Turecki G Brent DA. Suicide and suicidal behaviour. Lancet. (2016) 387:1227–39. doi: 10.1016/S0140-6736(15)00234-2, PMID: 26385066 PMC5319859

[10] Schmaal L van Harmelen AL Chatzi V Lippard ETC Toenders YJ Averill LA. Imaging suicidal thoughts and behaviors: a comprehensive review of 2 decades of neuroimaging studies. Mol Psychiatry. (2020) 25:408–27. doi: 10.1038/s41380-019-0587-x, PMID: 31787757 PMC6974434

[11] Auerbach RP Pagliaccio D Allison GO Alqueza KL Alonso MF. Neural correlates associated with suicide and nonsuicidal self-injury in youth. Biol Psychiatry. (2021) 89:119–33. doi: 10.1016/j.biopsych.2020.06.002, PMID: 32782140 PMC7726029

[12] Williams JMG Williams M. Cry of pain: understanding suicide and the suicidal mind. London, England:Piatkus (2014)

[13] O’Connor RC Kirtley OJ. The integrated motivational-volitional model of suicidal behaviour. Philos Trans R Soc Lond Ser B Biol Sci. (2018) 373:20170268. doi: 10.1098/rstb.2017.0268, PMID: 30012735 PMC6053985

[14] Mann JJ Waternaux C Haas GL Malone KM. Toward a clinical model of suicidal behavior in psychiatric patients. Am J Psychiatry. (1999) 156:181–9. doi: 10.1176/ajp.156.2.181, PMID: 9989552

[15] Brådvik L. Suicide risk and mental disorders. Int J Environ Res Public Health. (2018) 15:2028. doi: 10.3390/ijerph15092028, PMID: 30227658 PMC6165520

[16] Brown VM Wilson J Hallquist MN Szanto K Dombrovski AY. Ventromedial prefrontal value signals and functional connectivity during decision-making in suicidal behavior and impulsivity. Neuropsychopharmacology. (2020) 45:1034–41. doi: 10.1038/s41386-020-0632-0, PMID: 32035425 PMC7162923

[17] Jollant F Lawrence NS Giampietro V Brammer MJ Fullana MA Drapier D. Orbitofrontal cortex response to angry faces in men with histories of suicide attempts. Am J Psychiatr. (2008) 165:740–8. doi: 10.1176/appi.ajp.2008.07081239, PMID: 18346998

[18] Li H Chen Z Gong Q Jia Z. Voxel-wise meta-analysis of task-related brain activation abnormalities in major depressive disorder with suicide behavior. Brain Imaging Behav. (2020) 14:1298–308. doi: 10.1007/s11682-019-00045-3, PMID: 30790165

[19] Olié E Jollant F Deverdun J De Champfleur NM Cyprien F Le Bars E. The experience of social exclusion in women with a history of suicidal acts: a neuroimaging study. Sci Rep. (2017) 7:89. doi: 10.1038/s41598-017-00211-x 28273888 PMC5428048

[20] Cáceda R James GA Stowe ZN Delgado PL Kordsmeier N Kilts CD. The neural correlates of low social integration as a risk factor for suicide. Eur Arch Psychiatry Clin Neurosci. (2020) 270:619–31. doi: 10.1007/s00406-019-00990-6, PMID: 30903270 PMC6756996

[21] Miller AB McLaughlin KA Busso DS Brueck S Peverill M Sheridan MA. Neural correlates of emotion regulation and adolescent suicidal ideation. Biol Psychiatry Cogn Neurosci Neuroimaging. (2018) 3:125–32. doi: 10.1016/j.bpsc.2017.08.008, PMID: 29529407 PMC5851479

[22] Pan LA Hassel S Segreti AM Nau SA Brent DA Phillips ML. Differential patterns of activity and functional connectivity in emotion processing neural circuitry to angry and happy faces in adolescents with and without suicide attempt. Psychol Med. (2013) 43:2129–42. doi: 10.1017/S0033291712002966, PMID: 23298821

[23] Ai H van Tol MJ Marsman JBC Veltman DJ Ruhé HG van der Wee NJA. Differential relations of suicidality in depression to brain activation during emotional and executive processing. J Psychiatr Res. (2018) 105:78–85. doi: 10.1016/j.jpsychires.2018.08.018, PMID: 30212727

[24] Alarcón G Sauder M Teoh JY Forbes EE Quevedo K. Amygdala functional connectivity during self-face processing in depressed adolescents with recent suicide attempt. J Am Acad Child Adolesc Psychiatry. (2019) 58:221–31. doi: 10.1016/j.jaac.2018.06.036 30738549 PMC6492541

[25] Malhi GS Das P Outhred T Gessler D John Mann J Bryant R. Cognitive and emotional impairments underpinning suicidal activity in patients with mood disorders: an fMRI study. Acta Psychiatr Scand. (2019) 139:454–63. doi: 10.1111/acps.13022, PMID: 30865285

[26] Just MA Pan L Cherkassky VL McMakin DL Cha C Nock MK. Machine learning of neural representations of suicide and emotion concepts identifies suicidal youth. Nat Hum Behav. (2017) 1:911–9. doi: 10.1038/s41562-017-0234-y, PMID: 29367952 PMC5777614

[27] Richard-Devantoy S Ding Y Lepage M Turecki G Jollant F. Cognitive inhibition in depression and suicidal behavior: a neuroimaging study. Psychol Med. (2016) 46:933–44. doi: 10.1017/S0033291715002421, PMID: 26670261

[28] van Velzen LS Dauvermann MR Colic L Villa LM Savage HS Toenders YJ. Structural brain alterations associated with suicidal thoughts and behaviors in young people: results across 21 international studies from the ENIGMA suicidal thoughts and behaviours consortium. Med Rxiv. (2021). doi: 10.1038/s41380-022-01734-0 PMC9734039 36071108

[29] Bajaj S Adhikari BM Friston KJ Dhamala M. Bridging the gap: dynamic causal modeling and granger causality analysis of resting state functional magnetic resonance imaging. Brain Connect. (2016) 6:652–61. doi: 10.1089/brain.2016.0422, PMID: 27506256

[30] Bajaj S Adhikari BM Dhamala M. Higher frequency network activity flow predicts lower frequency node activity in intrinsic low-frequency BOLD fluctuations. PLoS One. (2013) 8:e64466. doi: 10.1371/journal.pone.0064466, PMID: 23691225 PMC3655147

[31] Bajaj S Raikes AC Razi A Miller MA Killgore WDS. Blue-light therapy strengthens resting-state effective connectivity within default-mode network after mild TBI. J Cent Nerv Syst Dis. (2021) 13:11795735211015076. doi: 10.1177/11795735211015076, PMID: 34104033 PMC8145607

[32] Uddin LQ Yeo BTT Spreng RN. Towards a universal taxonomy of macro-scale functional human brain networks. Brain Topogr. (2019) 32:926–42. doi: 10.1007/s10548-019-00744-6, PMID: 31707621 PMC7325607

[33] Qiu H Cao B Cao J Li X Chen J Wang W. Resting-state functional connectivity of the anterior cingulate cortex in young adults depressed patients with and without suicidal behavior. Behav Brain Res. (2020) 384:112544. doi: 10.1016/j.bbr.2020.112544, PMID: 32035184

[34] Du L Zeng J Liu H Tang D Meng H Li Y. Fronto-limbic disconnection in depressed patients with suicidal ideation: a resting-state functional connectivity study. J Affect Disord. (2017) 215:213–7. doi: 10.1016/j.jad.2017.02.027, PMID: 28340447

[35] Yang Y Chattun MR Yan R Zhao K Chen Y Zhu R. Atrophy of right inferior frontal orbital gyrus and frontoparietal functional connectivity abnormality in depressed suicide attempters. Brain Imaging Behav. (2020) 14:2542–52. doi: 10.1007/s11682-019-00206-4, PMID: 32157476

[36] Stange JP Jenkins LM Pocius S Kreutzer K Bessette KL Deldonno SR. Using resting-state intrinsic network connectivity to identify suicide risk in mood disorders. Psychol Med. (2019) 50:2324–34. doi: 10.1017/S0033291719002356 31597581 PMC7368462

[37] Cao J Chen X Chen J Ai M Gan Y Wang W. Resting-state functional MRI of abnormal baseline brain activity in young depressed patients with and without suicidal behavior. J Affect Disord. (2016) 205:252–63. doi: 10.1016/j.jad.2016.07.002, PMID: 27467529

[38] Cao J Chen X Chen J Ai M Gan Y He J. The association between resting state functional connectivity and the trait of impulsivity and suicidal ideation in young depressed patients with suicide attempts. Front Psych. (2021) 12:567976. doi: 10.3389/fpsyt.2021.567976, PMID: 34393836 PMC8355430

[39] Zhang R Zhang L Wei S Wang P Jiang X Tang Y. Increased amygdala-paracentral lobule/precuneus functional connectivity associated with patients with mood disorder and suicidal behavior. Front Hum Neurosci. (2020) 14:585664. doi: 10.3389/fnhum.2020.611008, PMID: 33519398 PMC7843440

[40] Kang SG Na KS Choi JW Kim JH Son YD Lee YJ. Resting-state functional connectivity of the amygdala in suicide attempters with major depressive disorder. Prog Neuro-Psychopharmacol Biol Psychiatry. (2017) 77:222–7. doi: 10.1016/j.pnpbp.2017.04.029 28445688

[41] Wei S Chang M Zhang R Jiang X Wang F Tang Y. Amygdala functional connectivity in female patients with major depressive disorder with and without suicidal ideation. Ann General Psychiatry. (2018) 17:37. doi: 10.1186/s12991-018-0208-0, PMID: 30214465 PMC6134510

[42] Cao J Ai M Chen X Chen J Wang W Kuang L. Altered resting-state functional network connectivity is associated with suicide attempt in young depressed patients. Psychiatry Res. (2020) 285:112713. doi: 10.1016/j.psychres.2019.112713, PMID: 31810745

[43] Shu Y Kuang L Huang Q He L. Fractional amplitude of low-frequency fluctuation (fALFF) alterations in young depressed patients with suicide attempts after cognitive behavioral therapy and antidepressant medication cotherapy: a resting-state fMRI study. J Affect Disord. (2020) 276:822–8. doi: 10.1016/j.jad.2020.07.038, PMID: 32738667

[44] Zhang S Chen JM Kuang L Cao J Zhang H Ai M. Association between abnormal default mode network activity and suicidality in depressed adolescents. BMC Psychiatry. (2016) 16:337. doi: 10.1186/s12888-016-1047-7 27688124 PMC5041526

[45] Chen V Chou Y-S Tsai Y-H Huang Y-C McIntyre RS Weng J-C. Resting-state functional connectivity and brain network abnormalities in depressive patients with suicidal ideation. Brain Topogr. (2021) 34:234–44. doi: 10.1007/s10548-020-00817-x, PMID: 33420533

[46] Barredo J Aiken E Vant Wout-Frank M Greenberg BD Carpenter LL Philip NS. Neuroimaging correlates of suicidality in decision-making circuits in posttraumatic stress disorder. Front Psychiatry. (2019) 10:44. doi: 10.3389/fpsyt.2019.00044 30809160 PMC6379274

[47] Lee S Lee SM Kang WS Jahng GH Ryu CW Park JK. Altered resting-state functional connectivity in depressive disorder patients with suicidal attempts. Neurosci Lett. (2019) 696:174–8. doi: 10.1016/j.neulet.2018.12.037, PMID: 30593872

[48] Schreiner MW Klimes-Dougan B Cullen KR. Neural correlates of suicidality in adolescents with major depression: resting-state functional connectivity of the precuneus and posterior cingulate cortex. Suicide Life Threat Behav. (2019) 49:899–913. doi: 10.1111/sltb.12471, PMID: 29756354

[49] Kim K Kim SW Myung W Han CE Fava M Mischoulon D. Reduced orbitofrontal-thalamic functional connectivity related to suicidal ideation in patients with major depressive disorder. Sci Rep. (2017) 7:15772. doi: 10.1038/s41598-017-15926-0 29150619 PMC5693996

[50] Gosnell SN Fowler JC Salas R. Classifying suicidal behavior with resting-state functional connectivity and structural neuroimaging. Acta Psychiatr Scand. (2019) 140:20–9. doi: 10.1111/acps.13029, PMID: 30929253

[51] Dai Z Shen X Tian S Yan R Wang H Wang X. Gradually evaluating of suicidal risk in depression by semi-supervised cluster analysis on resting-state fMRI. Brain Imaging Behav. (2020) 15:2149–58. doi: 10.1007/s11682-020-00410-7 33151465

[52] Stumps A Jagger-Rickels A Rothlein D Amick M Park H Evans T. Connectome-based functional connectivity markers of suicide attempt. J Affect Disord. (2020). 430–40. doi: 10.1016/j.jad.2020.11.061 33549365

[53] Chase HW Segreti AM Keller TA Cherkassky VL Just MA Pan LA. Alterations of functional connectivity and intrinsic activity within the cingulate cortex of suicidal ideators. J Affect Disord. (2017) 212:78–85. doi: 10.1016/j.jad.2017.01.013, PMID: 28157550 PMC5358995

[54] Serafini G Pardini M Pompili M Girardi P Amore M. Understanding suicidal behavior: The contribution of recent resting-state fMRI techniques. Front Psychiatry. (2016) 7:69. doi: 10.3389/fpsyt.2016.00069 27148097 PMC4835442

[55] Hwang JP Lee TW Tsai SJ Chen TJ Yang CH Lirng JF. Cortical and subcortical abnormalities in late-onset depression with history of suicide attempts investigated with MRI and voxel-based morphometry. J Geriatr Psychiatry Neurol. (2010) 23:171–84. doi: 10.1177/0891988710363713, PMID: 20430976

[56] Wagner G Schultz CC Koch K Schachtzabel C Sauer H Schlösser RG. Prefrontal cortical thickness in depressed patients with high-risk for suicidal behavior. J Psychiatr Res. (2012) 46:1449–55. doi: 10.1016/j.jpsychires.2012.07.013, PMID: 22868048

[57] Huber RS McGlade EC Legarreta M Subramaniam P Renshaw PF Yurgelun-Todd DA. Cingulate white matter volume and associated cognitive and behavioral impulsivity in veterans with a history of suicide behavior. J Affect Disord. (2021) 281:117–24. doi: 10.1016/j.jad.2020.11.126, PMID: 33316716

[58] Wang P Zhang R Jiang X Wei S Wang F Tang Y. Gray matter volume alterations associated with suicidal ideation and suicide attempts in patients with mood disorders. Ann General Psychiatry. (2020) 19:69. doi: 10.1186/s12991-020-00318-y PMC7727241 33302965

[59] Ding Y Lawrence N Olie E Cyprien F le Bars E Bonafe A. Prefrontal cortex markers of suicidal vulnerability in mood disorders: a model-based structural neuroimaging study with a translational perspective. Transl Psychiatry. (2015) 5:e516. doi: 10.1038/tp.2015.1, PMID: 25710122 PMC4445751

[60] Fan S Lippard ETC Sankar A Wallace A Johnston JAY Wang F. Gray and white matter differences in adolescents and young adults with prior suicide attempts across bipolar and major depressive disorders. J Affect Disord. (2019) 245:1089–97. doi: 10.1016/j.jad.2018.11.095, PMID: 30699851 PMC6903411

[61] Lippard ETC Johnston JAY Spencer L Quatrano S Fan S Sankar A. Preliminary examination of gray and white matter structure and longitudinal structural changes in frontal systems associated with future suicide attempts in adolescents and young adults with mood disorders. J Affect Disord. (2019) 245:1139–48. doi: 10.1016/j.jad.2018.11.097, PMID: 30699858 PMC6487887

[62] Segreti AM Chase HW Just M Brent D Pan L. Cortical thickness and volume reductions in young adults with current suicidal ideation. J Affect Disord. (2019) 245:126–9. doi: 10.1016/j.jad.2018.10.081, PMID: 30388554

[63] Bajaj S Raikes AC Smith R Vanuk JR Killgore WDS. The role of prefrontal cortical surface area and volume in preclinical suicidal ideation in a non-clinical sample. Front Psychiatry. (2019) 10:445. doi: 10.3389/fpsyt.2019.00445 31312146 PMC6613495

[64] Kang SG Cho SE Na KS Lee JS Joo SW Cho SJ. Differences in brain surface area and cortical volume between suicide attempters and non-attempters with major depressive disorder. Psychiatry Res Neuroimaging. (2020) 297:111032. doi: 10.1016/j.pscychresns.2020.111032 32028105

[65] Harenski CL Harenski KA Calhoun VD Kiehl KA. Source-based morphometry reveals gray matter differences related to suicidal behavior in criminal offenders. Brain Imaging Behav. (2020) 14:1–9. doi: 10.1007/s11682-018-9957-2, PMID: 30215220 PMC6447455

[66] Kang W Shin JH Han KM Kim A Kang Y Kang J. Local shape volume alterations in subcortical structures of suicide attempters with major depressive disorder. Hum Brain Mapp. (2020) 41:4925–34. doi: 10.1002/hbm.25168, PMID: 32804434 PMC7643352

[67] Gosnell SN Velasquez KM Molfese DL Molfese PJ Madan A Fowler JC. Prefrontal cortex, temporal cortex, and hippocampus volume are affected in suicidal psychiatric patients. Psychiatry Res Neuroimaging. (2016) 256:50–6. doi: 10.1016/j.pscychresns.2016.09.005, PMID: 27685801 PMC9694115

[68] Chen F Bertelsen AB Holm IE Nyengaard JR Rosenberg R Dorph-Petersen KA. Hippocampal volume and cell number in depression, schizophrenia, and suicide subjects. Brain Res. (2020) 1727:146546. doi: 10.1016/j.brainres.2019.146546, PMID: 31715144

[69] Jollant F Wagner G Richard-Devantoy S Köhler S Bär KJ Turecki G. Neuroimaging-informed phenotypes of suicidal behavior: a family history of suicide and the use of a violent suicidal means. Transl Psychiatry. (2018) 8:120. doi: 10.1038/s41398-018-0170-2 29921964 PMC6008434

[70] Ho TC Cichocki AC Gifuni AJ Camacho MC Ordaz SJ Singh MK. Reduced dorsal striatal gray matter volume predicts implicit suicidal ideation in adolescents. Soc Cogn Affect Neurosci. (2018) 13:1215–24. doi: 10.1093/scan/nsy089, PMID: 30256980 PMC6234322

[71] Ho TC Teresi GI Ojha A Walker JC Kirshenbaum JS Singh MK. Smaller caudate gray matter volume is associated with greater implicit suicidal ideation in depressed adolescents. J Affect Disord. (2021) 278:650–7. doi: 10.1016/j.jad.2020.09.046, PMID: 33039875 PMC9386733

[72] Pan LA Ramos L Segreti A Brent DA Phillips ML. Right superior temporal gyrus volume in adolescents with a history of suicide attempt. Br J Psychiatry. (2015) 206:339–40. doi: 10.1192/bjp.bp.114.151316, PMID: 25497300 PMC4381193

[73] Vidal-Ribas P Janiri D Doucet GE Pornpattananangkul N Nielson DM Frangou S. Multimodal neuroimaging of suicidal thoughts and behaviors in a U.S. population-based sample of school-age children. Am J Psychiatr. (2021) 178:321–32. doi: 10.1176/appi.ajp.2020.20020120, PMID: 33472387 PMC8016742

[74] McLellan Q Wilkes TC Swansburg R Jaworska N Langevin LM Mac Master FP. History of suicide attempt and right superior temporal gyrus volume in youth with treatment-resistant major depressive disorder. J Affect Disord. (2018) 239:291–4. doi: 10.1016/j.jad.2018.07.030, PMID: 30031248

[75] Peng H Wu K Li J Qi H Guo S Chi M. Increased suicide attempts in young depressed patients with abnormal temporal-parietal-limbic gray matter volume. J Affect Disord. (2014) 165:69–73. doi: 10.1016/j.jad.2014.04.046, PMID: 24882180

[76] Lee YJ Kim S Gwak AR Kim SJ Kang SG Na KS. Decreased regional gray matter volume in suicide attempters compared to suicide non-attempters with major depressive disorders. Compr Psychiatry. (2016) 67:59–65. doi: 10.1016/j.comppsych.2016.02.013, PMID: 27095336

[77] Campos AI Thompson PM Veltman DJ Pozzi E van Veltzen LS Jahanshad N. Brain correlates of suicide attempt in 18, 925 participants across 18 international cohorts. Biol Psychiatry. (2021) 90:243–52. doi: 10.1016/j.biopsych.2021.03.015, PMID: 34172278 PMC8324512

[78] Sarkinaite M Gleizniene R Adomaitiene V Dambrauskiene K Raskauskiene N Steibliene V. Volumetric MRI analysis of brain structures in patients with history of first and repeated suicide attempts: a cross sectional study. Diagnostics. (2021) 11:488. doi: 10.3390/diagnostics11030488. 33801896 PMC8000590

[79] Raichle ME. The brain’s default mode network. Annu Rev Neurosci. (2015) 38:433–47. doi: 10.1146/annurev-neuro-071013-014030, PMID: 25938726

[80] Chand GB Dhamala M. The salience network dynamics in perceptual decision-making. Neuroimage. (2016) 134:85–93. doi: 10.1016/j.neuroimage.2016.04.018, PMID: 27079535

[81] Chand GB Dhamala M. Interactions among the brain default-mode, salience, and central-executive networks during perceptual decision-making of moving dots. Brain Connect. (2015) 6:249–54. doi: 10.1089/brain.2015.0379 26694702

[82] Fischl B Sereno MI Dale AM. Cortical surface-based analysis. II: inflation, flattening, and a surface-based coordinate system. NeuroImage. (1999) 9:195–207. doi: 10.1006/nimg.1998.0396, PMID: 9931269

[83] Dale AM Fischl B Sereno MI. Cortical surface-based analysis. I. Segmentation and surface reconstruction. Neuroimage. (1999) 9:179–94. doi: 10.1006/nimg.1998.0395, PMID: 9931268

[84] Yeo BT Krienen FM Sepulcre J Sabuncu MR Lashkari D Hollinshead M. The organization of the human cerebral cortex estimated by intrinsic functional connectivity. J Neurophysiol. (2011) 106:1125–65. doi: 10.1152/jn.00338.2011, PMID: 21653723 PMC3174820

[85] Provenzale JM Isaacson J Chen S Stinnett S Liu C. Correlation of apparent diffusion coefficient and fractional anisotropy values in the developing infant brain. AJR Am J Roentgenol. (2010) 195:W456–62. doi: 10.2214/AJR.10.4886 21098179 PMC3640803

[86] Cheng H Wang Y Sheng J Kronenberger WG Mathews VP Hummer TA. Characteristics and variability of structural networks derived from diffusion tensor imaging. NeuroImage. (2012) 61:1153–64. doi: 10.1016/j.neuroimage.2012.03.036, PMID: 22450298 PMC3500617

[87] Huang HQ Ding MZ. Linking functional connectivity and structural connectivity quantitatively: a comparison of methods. Brain Connect. (2016) 6:99–108. doi: 10.1089/brain.2015.0382, PMID: 26598788 PMC4779964

[88] Jia Z Huang X Wu Q Zhang T Lui S Zhang J. High-field magnetic resonance imaging of suicidality in patients with major depressive disorder. Am J Psychiatr. (2010) 167:1381–90. doi: 10.1176/appi.ajp.2010.09101513, PMID: 20843871

[89] Jia Z Wang Y Huang X Kuang W Wu Q Lui S. Impaired frontothalamic circuitry in suicidal patients with depression revealed by diffusion tensor imaging at 3.0 T. J Psychiatry Neurosci. (2014) 39:170–7. doi: 10.1503/jpn.130023, PMID: 24119793 PMC3997602

[90] Kim B Oh J Kim MK Lee S Tae WS Kim CM. White matter alterations are associated with suicide attempt in patients with panic disorder. J Affect Disord. (2015) 175:139–46. doi: 10.1016/j.jad.2015.01.001, PMID: 25617685

[91] Myung W Han CE Fava M Mischoulon D Papakostas GI Heo JY. Reduced frontal-subcortical white matter connectivity in association with suicidal ideation in major depressive disorder. Transl Psychiatry. (2016) 6:e835. doi: 10.1038/tp.2016.110, PMID: 27271861 PMC4931608

[92] Olvet DM Peruzzo D Thapa-Chhetry B Sublette ME Sullivan GM Oquendo MA. A diffusion tensor imaging study of suicide attempters. J Psychiatr Res. (2014) 51:60–7. doi: 10.1016/j.jpsychires.2014.01.002, PMID: 24462041 PMC4060601

[93] Wei S Womer FY Edmiston EK Zhang R Jiang X Wu F. Structural alterations associated with suicide attempts in major depressive disorder and bipolar disorder: a diffusion tensor imaging study. Prog Neuro-Psychopharmacol Biol Psychiatry. (2020) 98:109827. doi: 10.1016/j.pnpbp.2019.109827 31778758

[94] Bijttebier S Caeyenberghs K van den Ameele H Achten E Rujescu D Titeca K. The vulnerability to suicidal behavior is associated with reduced connectivity strength. Front Hum Neurosci. (2015) 9:632. doi: 10.3389/fnhum.2015.00632, PMID: 26648857 PMC4663245

[95] Hwang J Legarreta M Bueler CE DiMuzio J McGlade E Lyoo IK. Increased efficiency of brain connectivity networks in veterans with suicide attempts. Neuroimage Clin. (2018) 20:318–26. doi: 10.1016/j.nicl.2018.04.021, PMID: 30105203 PMC6086217

[96] Chen V Kao C-J Tsai Y-H McIntyre RS Weng J-C. Mapping brain microstructure and network alterations in depressive patients with suicide attempts using generalized Q-sampling MRI. J Pers Med. (2021) 11:174. doi: 10.3390/jpm11030174 33802354 PMC7998726

[97] Chen VC-H Kao C-J Tsai Y-H Cheok MT McIntyre RS Weng J-C. Assessment of disrupted brain structural connectome in depressive patients with suicidal ideation using generalized Q-sampling MRI. Front Hum Neurosci. (2021) 15:711731. doi: 10.3389/fnhum.2021.711731, PMID: 34512298 PMC8430248

[98] Beck JS. Cognitive behavior therapy: basics and beyond. New York: The Guilford Press (2011).

[99] Desmyter S Bijttebier S Heeringen K. The role of neuroimaging in our understanding of the suicidal brain. CNS Neurol Disord Drug Targets. (2013) 12:921–9. doi: 10.2174/18715273113129990093, PMID: 24040805

[100] Zhang H Chen Z Jia Z Gong Q. Dysfunction of neural circuitry in depressive patients with suicidal behaviors: a review of structural and functional neuroimaging studies. Prog Neuro-Psychopharmacol Biol Psychiatry. (2014) 53:61–6. doi: 10.1016/j.pnpbp.2014.03.002 24632395

[101] Martin PC Zimmer TJ Pan LA. Magnetic resonance imaging markers of suicide attempt and suicide risk in adolescents. CNS Spectr. (2014) 20:355–8. doi: 10.1017/S1092852915000048 25907463

[102] Sher L. Functional magnetic resonance imaging in studies of the neurobiology of suicidal behavior in adolescents with alcohol use disorders. Int J Adolesc Med Health. (2007) 19:11–8. doi: 10.1515/IJAMH.2007.19.1.11, PMID: 17458319

[103] van Heeringen K Bijttebier S Desmyter S Vervaet M Baeken C. Is there a neuroanatomical basis of the vulnerability to suicidal behavior? A coordinate-based meta-analysis of structural and functional MRI studies. Front Hum Neurosci. (2014) 8:824. doi: 10.3389/fnhum.2014.00824, PMID: 25374525 PMC4205829

[104] Desmyter S van Heeringen C Audenaert K. Structural and functional neuroimaging studies of the suicidal brain. Prog Neuro-Psychopharmacol Biol Psychiatry. (2011) 35:796–808. doi: 10.1016/j.pnpbp.2010.12.026 21216267

[105] Bani-Fatemi A Tasmim S Graff-Guerrero A Gerretsen P Strauss J Kolla N. Structural and functional alterations of the suicidal brain: an updated review of neuroimaging studies. Psychiatry Res Neuroimaging. (2018) 278:77–91. doi: 10.1016/j.pscychresns.2018.05.008, PMID: 29929763

[106] Olié E Ding Y Le Bars E de Champfleur NM Mura T Bonafé A. Processing of decision-making and social threat in patients with history of suicidal attempt: a neuroimaging replication study. Psychiatry Res Neuroimaging. (2015) 234:369–77. doi: 10.1016/j.pscychresns.2015.09.020, PMID: 26483212

[107] Simons LE Elman I Borsook D. Psychological processing in chronic pain: a neural systems approach. Neurosci Biobehav Rev. (2014) 39:61–78. doi: 10.1016/j.neubiorev.2013.12.006 24374383 PMC3944001

[108] Kross E Berman MG Mischel W Smith EE Wager TD. Social rejection shares somatosensory representations with physical pain. Proc Natl Acad Sci U S A. (2011) 108:6270–5. doi: 10.1073/pnas.1102693108, PMID: 21444827 PMC3076808

[109] Eisenberger NI. The pain of social disconnection: examining the shared neural underpinnings of physical and social pain. Nat Rev Neurosci. (2012) 13:421–34. doi: 10.1038/nrn3231, PMID: 22551663

[110] Olié E Courtet P. Interest of neuroimaging of social exclusion in suicide. J Neurosci Res. (2020) 98:581–7. doi: 10.1002/jnr.24314 30171628

[111] Friston KJ. Functional and effective connectivity: a review. Brain Connect. (2011) 1:13–36. doi: 10.1089/brain.2011.0008, PMID: 22432952

[112] Bajaj S Lamichhane B Adhikari BM Dhamala M. Amygdala mediated connectivity in perceptual decision-making of emotional facial expressions. Brain Connect. (2013) 3:386–97. doi: 10.1089/brain.2013.0145, PMID: 23705655

[113] Bajaj S Killgore WDS. Association between emotional intelligence and effective brain connectome: a large-scale spectral DCM study. NeuroImage. (2021) 229:117750. doi: 10.1016/j.neuroimage.2021.117750 33454407

[114] Bajaj S Housley SN Wu D Dhamala M James GA Butler AJ. Dominance of the unaffected hemisphere motor network and its role in the behavior of chronic stroke survivors. Front Hum Neurosci. (2016) 10:650. doi: 10.3389/fnhum.2016.00650, PMID: 28082882 PMC5186808

[115] Killgore WDS Vanuk JR Shane BR Weber M Bajaj S. A randomized, double-blind, placebo-controlled trial of blue wavelength light exposure on sleep and recovery of brain structure, function, and cognition following mild traumatic brain injury. Neurobiol Dis. (2020) 134:104679. doi: 10.1016/j.nbd.2019.104679, PMID: 31751607

[116] Bajaj S Butler AJ Drake D Dhamala M. Brain effective connectivity during motor-imagery and execution following stroke and rehabilitation. Neuroimage Clin. (2015) 8:572–82. doi: 10.1016/j.nicl.2015.06.006, PMID: 26236627 PMC4501560

[117] Bajaj S Butler AJ Drake D Dhamala M. Functional organization and restoration of the brain motor-execution network after stroke and rehabilitation. Front Hum Neurosci. (2015) 9:173. doi: 10.3389/fnhum.2015.00173, PMID: 25870557 PMC4378298

